# Comprehensive overview of heat management methods for enhancing photovoltaic thermal systems

**DOI:** 10.1016/j.isci.2024.110950

**Published:** 2024-09-14

**Authors:** Md Atiqur Rahman, Sanjay Kumar Gupta, Nurgali Akylbekov, Rakhmetulla Zhapparbergenov, S. M. Mozammil Hasnain, Rustem Zairov

**Affiliations:** 1 Department of Mechanical Engineering, Vignan’s Foundation for Science, Technology & Research (Deemed to be University), Vadlamudi, Guntur, Andhra Pradesh 522213, India; 2 Laboratory of Engineering Profile “Physical and Chemical Methods of Analysis”, Korkyt Ata Kyzylorda University, Aiteke bi Str. 29A, Kyzylorda 120014, Kazakhstan; 3 Marwadi University Research Center, Department of Mechanical Engineering, Faculty of Engineering & Technology, Marwadi University, Rajkot, Gujarat 360003, India; 4 Aleksander Butlerov Institute of Chemistry, Kazan Federal University, 1/29 Lobachevskogo Str., Kazan 420008, Russian Federation; 5 A.E. Arbuzov Institute of Organic and Physical Chemistry, Kazan Scientific Center, Russian Academy of Sciences, 8 Arbuzov str., 420088 Kazan, Russian Federation

**Keywords:** Applied sciences, Energy systems

## Abstract

The paper examines strategies to improve the efficiency of photovoltaic (PV) systems, which are challenged by high operating temperatures that reduce performance. It focuses on enhancing PV systems through the use of gallium arsenide (GaAs) thin films and reviews techniques like spectral beam splitting to boost efficiency, particularly in multi-junction PV receivers and hybrid collectors. The study also explores Photovoltaic-thermal (PVT) systems that combine PV cells with thermal absorbers, highlighting advanced absorber designs, mini/microchannels, and the use of polymers over traditional metals. Additionally, the incorporation of phase change materials (PCM) and nanofluids is discussed for their potential to improve thermal conductivity and storage. By synthesizing experimental and numerical research, the paper emphasizes the importance of these innovations in advancing PVT systems for sustainable energy production.

## Graphical abstract

**Figure:**
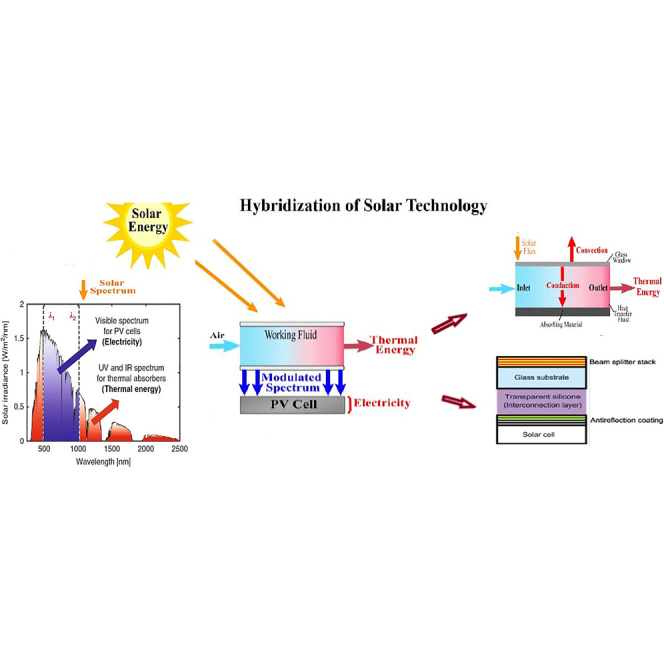

## Introduction

In the past few decades, the rising need for energy has run alongside advances in technology and industry.^1^ This increase has caused a greater dependence on fossil fuels as the primary energy source in different sectors.^2^

In addition to their limited supply, fossil fuels have played a significant role in emitting carbon. In 2023, there was a 1.1% rise in global CO_2_ emissions related to energy, amounting to an increase of 410 million Mt and reaching a new peak of 37.4 billion tonnes (Gt). This contrasts with the previous year’s increase of 490 Mt (1.3%) in 2022, worsening the issue of global warming.^3^ Consequently, continuous research endeavors seek to discover renewable energy options to supplant conventional commercial fuels. Among various renewable energy sources such as bioenergy, hydropower, wind, and geothermal, solar energy is a prominent contender in renewable energy advancement. This is particularly evident through the utilization of photovoltaic (PV) technology.^4^ Each year, the potential of solar energy amounts to roughly 4 million exajoules, establishing it as a better alternative energy option because of its abundance and minimized emissions (greenhouse gas).^5^

**Figure 1:**
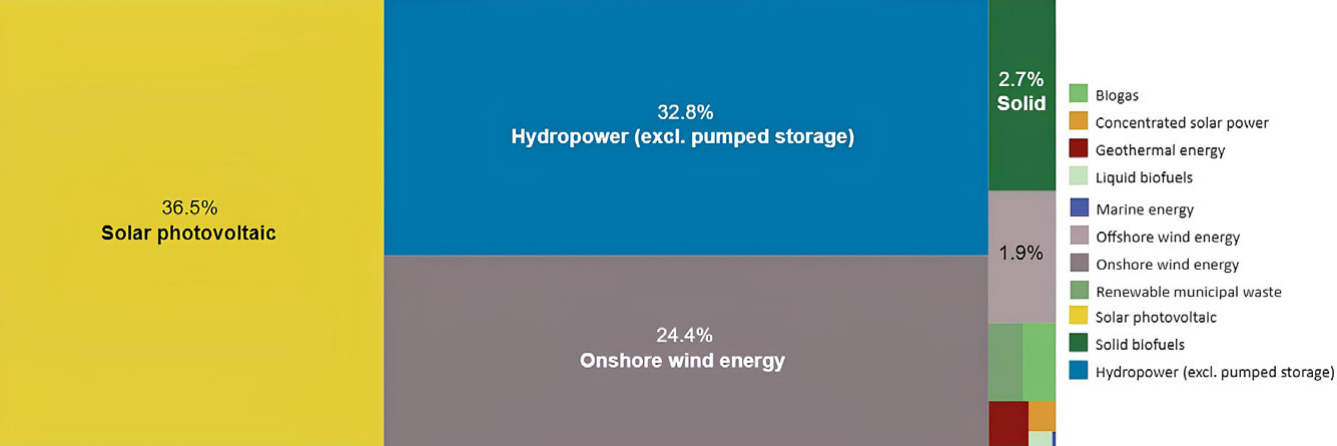
Latest global trends in renewable energy Reproduced with permission^6^ copyright 2011–2022 IRENA.

As per the IREA, PV is first in renewable energy sources, followed only by hydropower. In 2023, the capacity of PV rose to 36.5%, reflecting a 5.1% surge from the preceding year.^6^ As illustrated in Figure 1,^6^ electricity generation through PVS (photovoltaic system) has shown a consistent upward trend across all regions, indicating the increasing public attention and the expected ongoing surge in demand.

The adaptability of PV technology, as evidenced by its various uses, such as personal wearable gadgets, building systems, and transportation, continues to drive its needs.^7^

**Figure 2:**
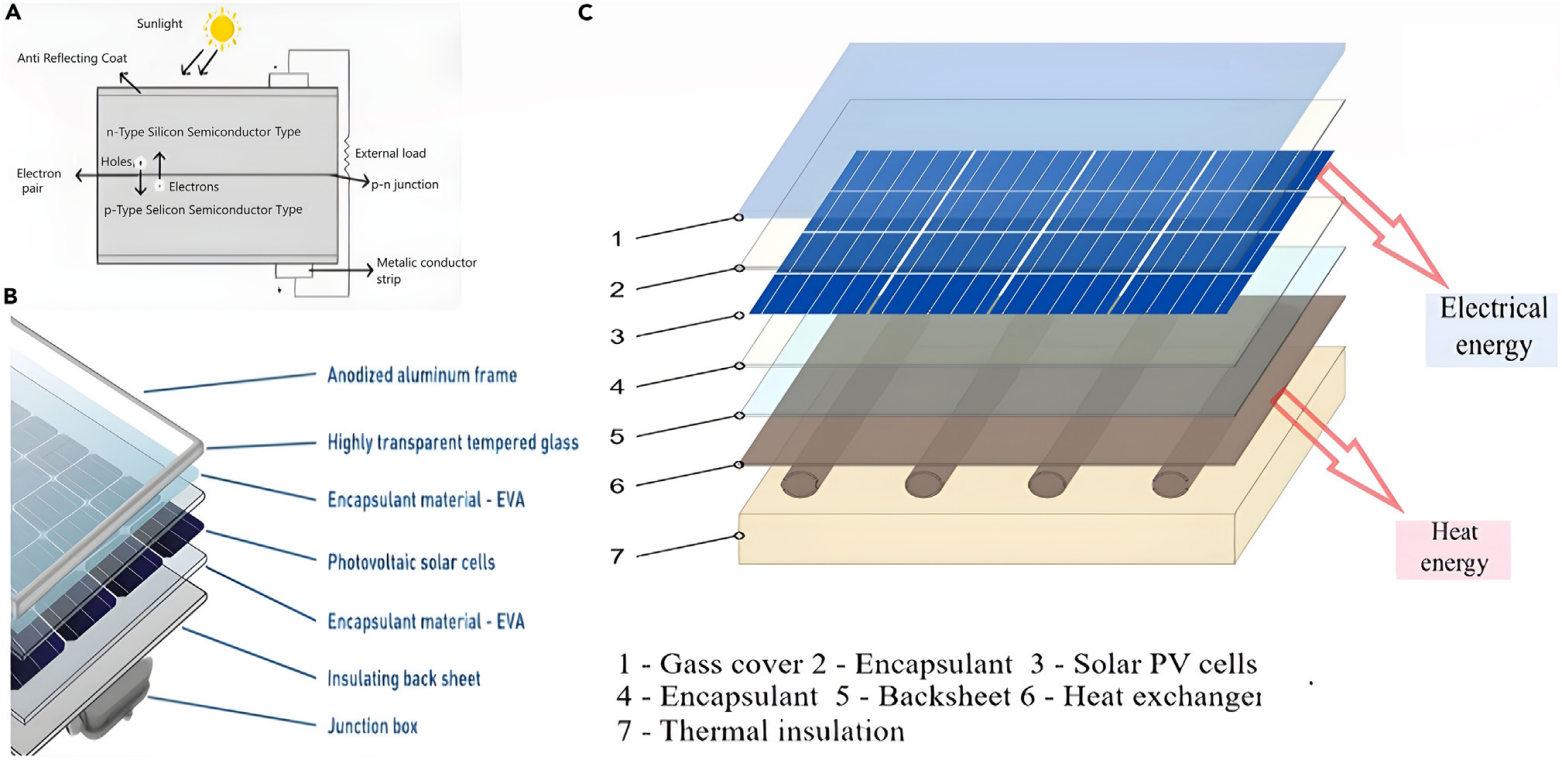
Different components of PV system (A) solar cell, (B) PV element, (C) PVT component. Reproduced with permission.^8^ Copyright 2020 wikimedia.

A typical PVS consists of multiple PV cells shielded by glass on the front and plastic on the rear, vacuum-sealed in a transparent polymer shown in Figure 2.^8^ Here’s a detailed look at each component^8^^,^^9^^,^^10^^,^^11^^,^^12^^,^^13^:

*PV Solar Cell:* these cells capture sunlight and convert it into electricity. They can be monocrystalline or polycrystalline, with size, color, and conversion efficiency being key technical characteristics.

*Front Glass:* this sturdy layer protects the module while maintaining transparency. It’s usually around 3.2mm thick and can have unique features like light-trapping patterns for enhanced efficiency.

*Back sheet:* made of plastic, this sheet isolates and protects cells from weather. It varies in thickness, color, and materials for added shielding or strength.

*Encapsulate Material*: the binder between layers, often ethylene vinyl acetate (EVA), ensures durability through a lamination process, impacting light transmission and UV resistance.

*Frame:* typically aluminum, the frame provides stability and facilitates module coupling, often sealed with silicone for moisture protection. Frameless or plastic-based alternatives are also available.

*Junction Box:* this box houses electrical connections, protection diodes, and cables for external connection. Quality considerations include material, sealing, and diode type, with emerging options like low-loss diodes and integrated micro-inverters showing promise despite current pricing limitations.

**Figure 3:**
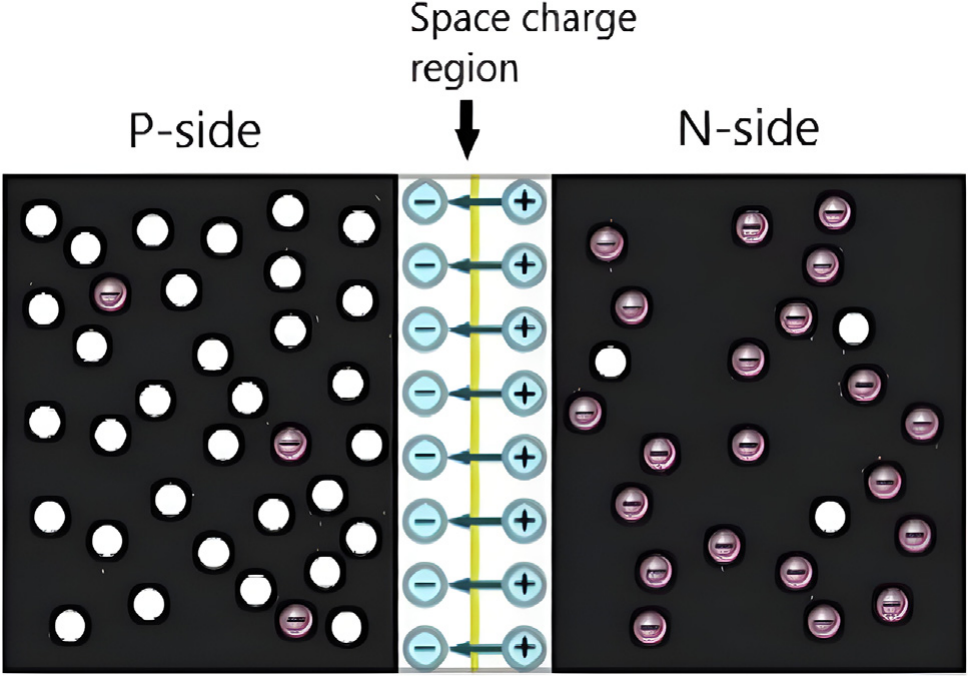
Semiconductor with p-n sections and generating the electric field in the space charge region Reproduced with permission.^15^ copyright 2022 MDPI.

The most crucial component of the PVT is the SC, a specialized semiconductor diode designed to convert sunlight into electrical power. A diode consists of a single crystal semiconductor, such as silicon, with one side doped with pentavalent impurities to create an n-type region and the other doped with trivalent impurities to form a p-type region. This doping process introduces additional mobile carriers, known as majority carriers, in each region. When the n-type and p-type materials are joined, electrons from the n-type region move into the p-type region, leaving behind positively charged donor atoms at the p-n junction near the n-type side. Similarly, the p-type region loses holes, resulting in a net of negatively charged acceptor atoms near the p-type side of the junction. This movement of electrons and holes creates what is identified as the diffusion current (I_diff_) and forms a depletion region where charge carriers are absent. An electric field generated in this depletion region pushes the electrons and holes out, preventing further movement of charge carriers,^14^ as illustrated in Figure 3.^15^

**Figure 4:**
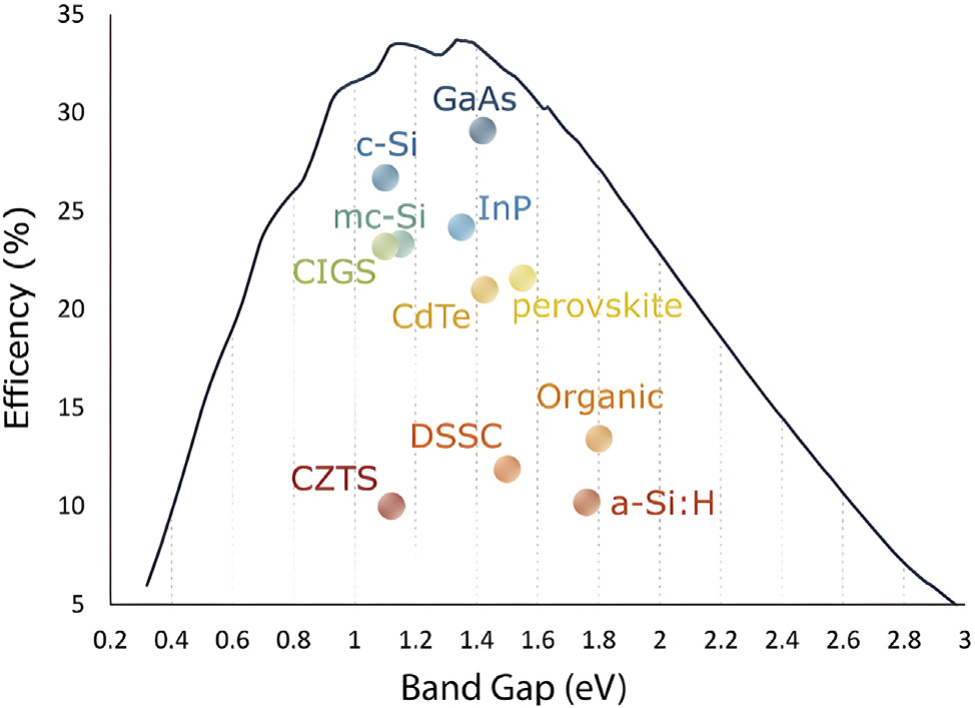
Solar PV band energy Reproduced with permission. Reproduced with permission^16^ copyright 2020 Cell Press.

Not all wavelengths of light are converted into electricity by PV cells. Commercial single-junction PV cells typically convert between 6% and 25% of light energy into electricity, with the rest lost as heat due to the semiconductor’s band-gap energy. For instance, crystalline silicon PV cells can utilize visible and some infrared light, but wavelengths outside this range contribute to heat rather than electrical power. This heating can raise the PV module’s temperature by up to 40°C above ambient, increasing the dark saturation current and reducing the cell’s electrical output by 0.2–0.5% for each 1°C surge in temperature. The PV material of various band gaps is revealed in Figure 4.^16^

**Figure 5:**
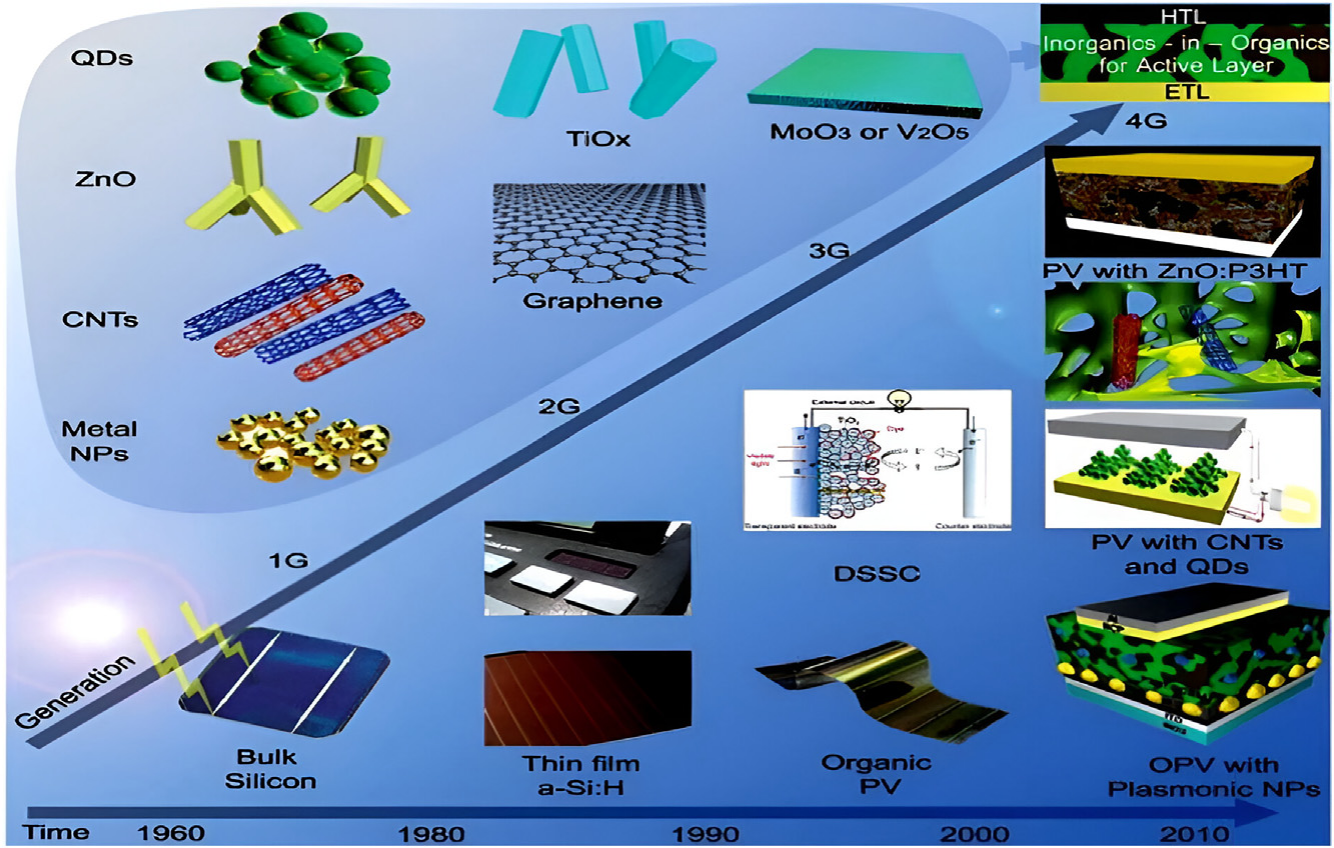
Chronology of the four generations of PV cells along with the materials used in each generation Reproduced with permission.^18^ copyright 2013 RSC.

The configuration of a PV cell is dictated by its structural design.^17^ According to the latest development in PVCs, the different generations of SC are characterized, as seen in Figure 5.^18^

Although there has been recent advancement in PV technologies to maximize efficiency, which includes modifying the solar cells themselves like 1st generation used CSi-SC, and 2^nd^ generation used thin-film technologies such as amorphous Si, Cu indium gallium di-selenide, and single-junction gallium arsenide (GaAs) cells.^19^ While CSi-PV cells are primarily utilized commercially due to their simple manufacturing process, other variations are still being researched. Furthermore, the enhancement of PV efficiency has included the adoption of solar trackers, capable of capturing sunlight during the day irrespective of *θ*,^20^ and hybrid systems, which integrate PV and other sources of energy (sustainable) to produce greater energy output.^21^

Despite these progressions, PVS typically attain an efficiency of a maximum 15% only, indicating that the remaining 85% of SE is untapped.^22^ To date, outdoor PVS exposure leads to overheating (a key component in thermal efficiency). The effectiveness of PVS systems declines by 0.4–0.5% with each 1°C rise in temperature resulting from increased solar radiation irradiance.^23^ Moreover, the temperature of solar cells can escalate due to dust accumulation on their surfaces.^24^ While routine dust elimination is crucial maintenance, it fails to prevent performance deterioration attributable to shading completely.^25^

Due to the factors mentioned, solar cells experience a rise in temperature during operation, particularly when exposed to concentrated sunlight. This temperature increase negatively impacts conversion efficiency: the open-circuit voltage and fill factor decrease with rising temperature, while the short-circuit current only slightly increases. For instance, the conversion efficiency of crystalline silicon (c-Si) cells drops by 0.004–0.005 per degree Celsius increase in temperature, whereas for III–V multi-junction cells, the decrease is between 0.001 and 0.003 per degree Celsius. Additionally, higher temperatures can reduce the lifespan of solar cell systems.

Two main approaches are typically employed to mitigate these temperature effects and enhance the efficiency of solar cell modules: spectral beam splitting (SBS) and waste heat recovery (WHR).

### Spectral beam splitting

A more recent technology to reduce the thermal load of PV cells is to use SBS, directing only part of the solar spectrum onto the PV receiver. This helps to place additional solar collectors to the beam directed away, which increases system efficiency.

SBS is a technique used to harness the entire spectrum of solar energy. In this process, sunlight is divided into two components based on photon energy.^26^^,^^27^ Shorter wavelengths with photon energies exceeding the semiconductor’s band gap are focused onto the solar cell’s surface and transformed into electrical energy.^28^ Meanwhile, the longer wavelengths, which have photon energies lower than the semiconductor’s band gap, are directed toward a heat sink, where they are converted into thermal energy.

**Figure 6:**
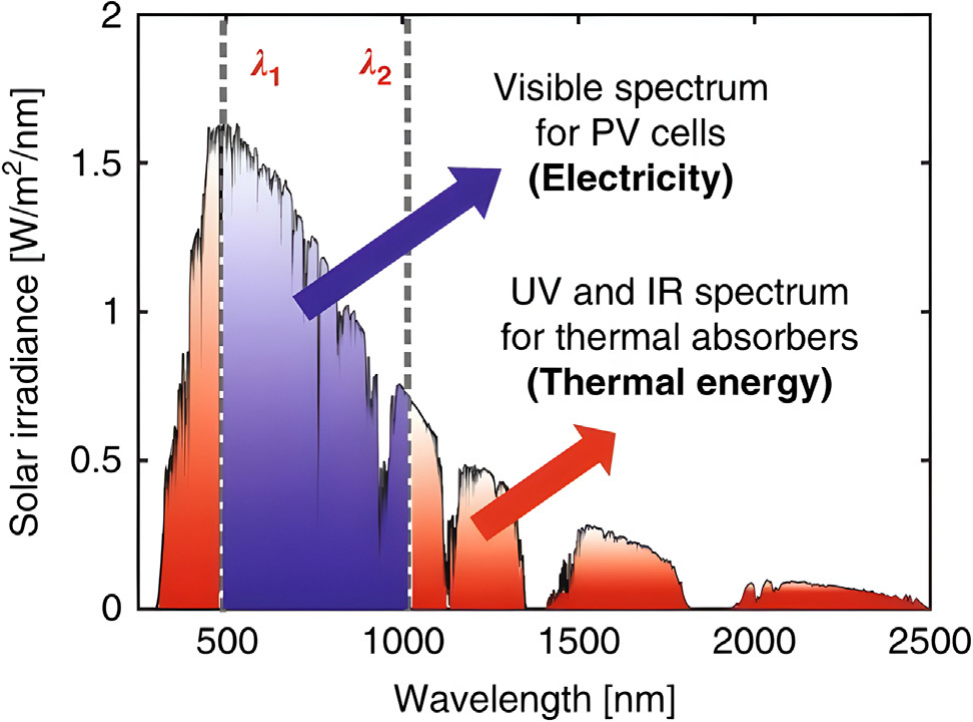
Splitting the solar spectrum into components for PV and thermal energy conversion Reproduced with permission^29^ copyright 2021 Springer Nature.

To optimize solar cell performance, it is advantageous to focus the solar spectrum on wavelengths that the PV cell can convert efficiently. The remaining spectrum should be redirected to another type of receiver, such as a thermal or chemical system or a different PV cell with a different band gap. This strategy is the basis of PV/thermal (PVT) hybrid solar systems, where incoming solar radiation is divided into PV and thermal utilization components, as depicted in Figure 6.^29^

Photothermal systems (PVT) efficiently convert solar energy into heat across the entire solar spectrum, as their performance mainly depends on the properties of the receiver’s window or coating. In contrast, PV systems are susceptible to the wavelength of light and work best with photons that match the PV cell’s band-gap energy. Photons with energy below this band gap pass through the cell without being absorbed and eventually turn into heat elsewhere in the cell. Photons with energy above the band gap are only partially used, with the excess energy also converting to heat.

High conversion efficiencies in PV cells can be achieved by matching different solar spectrum parts to cells with corresponding energy absorption ranges. Theoretically, up to 85% efficiencies are possible.^30^^,^^31^ This can be accomplished by either stacking cells in a series arrangement called cascade, tandem, or multijunction cells or placing them side-by-side in a parallel setup.

**Figure 7:**
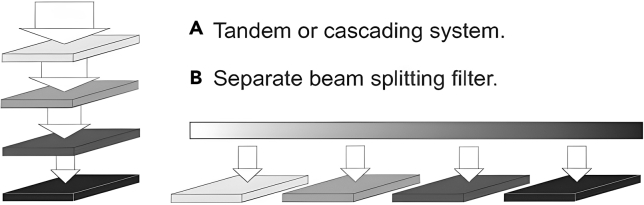
Two schemes for PV spectrum splitting (A) Tandem or cascading system. (B) Separate beam filter. Reproduced with permission^32^ Copyright 2004 Elsevier B.V.

In a tandem cell configuration (see Figure 7),^32^ sunlight first strikes the top cell with the most prominent band gap, which absorbs short-wavelength photons and generates high-energy electrons. The light that passes through this cell is then absorbed by a second cell with a smaller band gap, which captures longer wavelengths and generates lower-energy electrons. Multiple cells can be stacked to capture the entire solar spectrum.

Monolithic tandem cells use tunnel junctions to connect the cells in series, allowing the voltages to be combined. Alternatively, metal grids can interconnect the cells to achieve high voltage output. This setup produces higher voltage and lower current, reducing resistance losses at high concentrations. Tandem cells also require only one load and power-conditioning circuit, eliminating the need for separate optical filters. However, challenges include matching current and lattice structures and managing heat, as the top cell is cooled through its connection to the bottom cell.

An alternative approach, shown in Figure 7,^32^ involves placing cells in parallel. Here, a beam splitter filter divides the light into different spectral components, which are then directed onto separate cells with matching band gaps. This method allows each cell to be independently designed and manufactured, avoiding issues with substrate transparency or lattice mismatches. While this spectral splitting approach offers a slight theoretical efficiency advantage over cascading cells, the difference is minimal and may not be decisive.^33^

Current SSPVT collector concepts typically employ one of two spectral-splitting approaches based on selectively reflective or absorptive optical filters.

**Figure 8:**
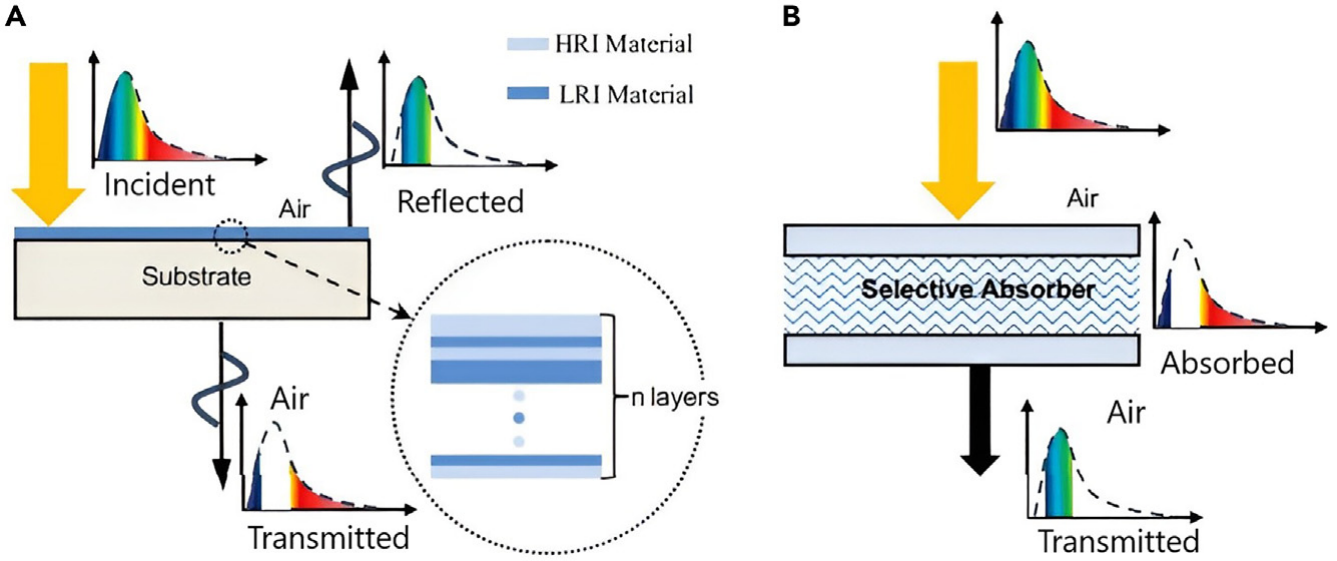
Spectral splitting mechanisms Reproduced with permission^37^ Copyright 2013 Elsevier Ltd. (A) Thin-film wave interference filter; High-RI and Low-RI. (B) Selective absorber (liquid or solid); (spectrum range available).

One approach involves holographic concentrators,^34^^,^^35^ which can split sunlight into different spectral bands and concentrate it. This method has proven effective in low-concentration solar collectors.^36^ One prominent technique uses thin-film interference optical filters, as illustrated in Figure 8A.^37^ These filters consist of multiple thin layers (ranging from a few nm to several hundred nm thick) made of non-absorptive dielectric materials with high refractive index contrasts, deposited on a transparent substrate to achieve specific optical properties (Figure 8A). These filters can be engineered to act as band-stop, band-pass, or edge filters. Macleod has provided a comprehensive theoretical framework for designing such filters.^38^

Rugate filters represent a distinct category of wave interference filters. Unlike multilayer filters, composed of discrete layers, rugate filters feature a refractive index that gradually varies throughout their thickness. This continuous variation endows rugate filters with enhanced mechanical strength and durability, making them more thermal stress-resistant than traditional multilayer filters. Additionally, there have been efforts to develop selective absorbing/transmitting filters, as depicted in Figure 8B.^37^ Peters et al. have provided a detailed review of the principles behind spectrally selective filtering techniques.^39^

**Figure 9:**
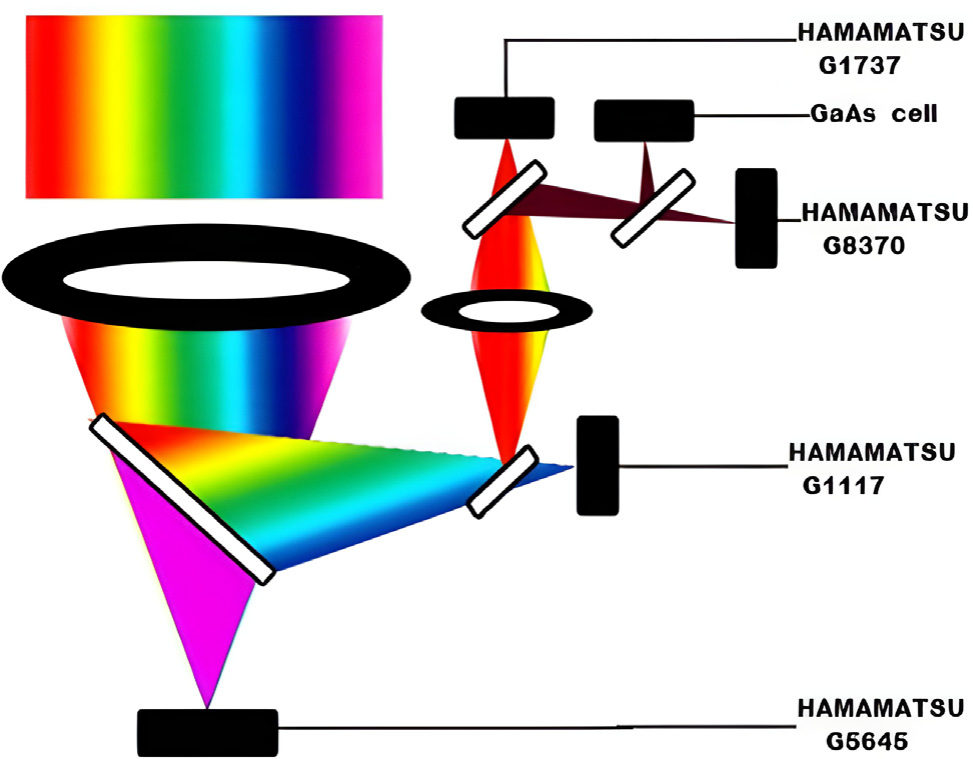
Concentrating PVS utilizing four dichroic mirrors dividing the spectrum into five bands Reproduced with permission^41^ Copyright 2012 Optical Society of America.

Theoretically, splitting the solar spectrum into multiple bands and directing each band to a specially designed cell can yield high solar conversion efficiencies. To test this theory, Zhao and Sheng separated the spectrum into three bands^40^ (excluding the integrated light separation in tandem cells) and five bands,^41^ as illustrated in Figure 9, respectively, using a series of selective mirrors, achieving 38% and 35.6% efficiencies at a 2.8x concentration for the three-band and five-band setups. The increased complexity associated with a higher number of bands decreased overall practical efficiency.

Xiong et al.^42^ indicated that for practical systems aiming to achieve high efficiencies, it is advisable not to divide the solar spectrum into too many bands due to the adverse effects of splitting losses on overall system efficiency. These splitting losses include the gradual transition between reflection and transmission, non-ideal cut-off wavelengths, and mirror reflection losses. For instance, if the transition range of the filter in the system increases from 10 nm to 100 nm, the system’s efficiency could decrease by 3%.^42^ Beyond splitting effects, the configuration of p–n junctions in systems similar to Figure 9 can also significantly impact total efficiency. Morki et al.^43^ demonstrated that using a configuration with a Si/Ge dual junction cell and a GaAs single junction cell can lead to a theoretical efficiency variation of 2.76% due to different p–n junction arrangements.

Non-ideal cut-off effects can arise due to factors such as the angle of incident light on the filter, the finite number of deposited layers, and inaccuracies during the filter fabrication.^44^ As illustrated in Figure 9,^41^ the angle of incidence on the spectral splitter in concentrating systems can vary considerably. Therefore, optimizing the geometric configuration to minimize or compensate for variations in the incident angle is crucial.

**Figure 10:**
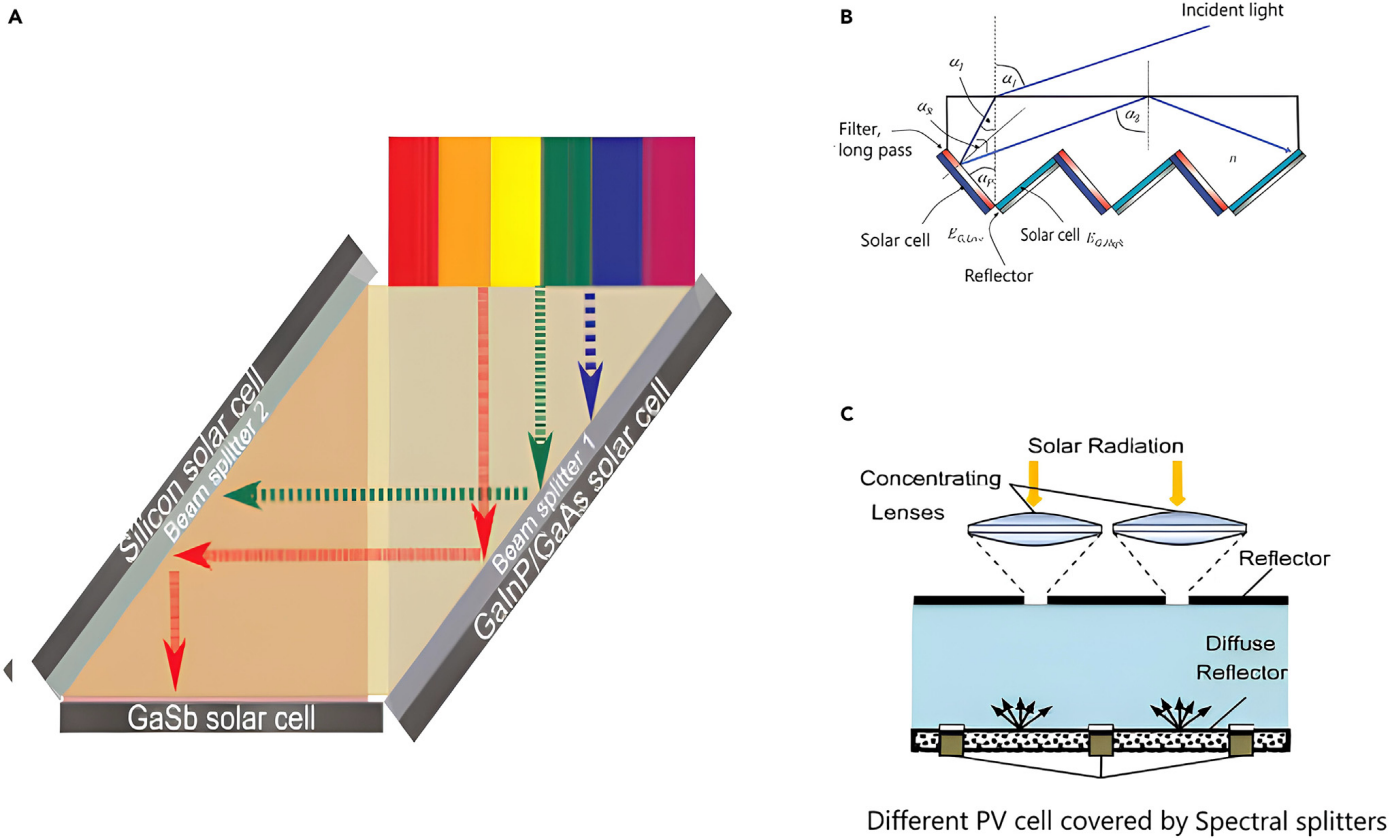
Recent advances in SBS technology used in PV Receivers (A) A light trap enhanced with two dichroic mirrors and three distinct SC arranged in a 45° parallele piped configuration. Beam splitter 1 allows wavelengths shorter than 850 nm to pass through, while beam splitter 2 permits wavelengths shorter than 1080 nm, reflecting all other wavelengths. Reproduced with permission^45^ Copyright 2010 John Wiley & Sons, Ltd. (B) A light-trapping receiver that employs total internal reflection, requiring only a single type of dichroic mirror (highlighted by the red rectangle). Long-wavelength light that passes through the high band gap cells is reflected back by a simple mirror positioned at the rear of these cells. Reproduced with permission^46^ Copyright 2012 Elsevier B.V. (C) A PV conversion system utilizing spectral splitting achieved with a set of concentrating lenses, a light trap, and small spectral mirrors. Reproduced with permission^36^ Copyright ©2008 Elsevier B.V.

Researchers have explored integrating light trapping with SBS to reduce reflection losses in optical devices within spectrally splitting systems. For example, Mitchell et al.^45^ proposed a configuration shown in Figure 10A,^45^ where reflection losses are minimized by ensuring that another component captures any light reflected from the surface of a beam splitter or cell.

In contrast, the design depicted in Figure 10B ^46^ focuses on collecting light from a larger aperture area. This setup employs total internal reflection to trap light within the optical receiver, using a single dichroic mirror to divide light between high and low-bandgap PV cells. When light encounters the long-pass filter, long-wavelength light is diffused through and absorbed by the low-bandgap cell, while short-wavelength light is reflected to the high-band gap cell. Conversely, when light reaches the high band gap cell, short-wavelength light is absorbed, and longer-wavelength light is diffused and then reflected by a highly reflective mirror back to the low band gap cell.

To leverage concentrated illumination in light trapping, Goetzberger et al.^47^ proposed the configurations shown in Figure 10C.^47^ Light is focused by lenses and directed through a small aperture into a light trap. Within the trap, radiation is diffused using Lambertian reflectors. Each solar cell inside the trap is covered with a band-pass mirror that transmits the optimized wavelength and reflects the rest. All other surfaces inside the trap are highly reflective.

**Figure 11:**
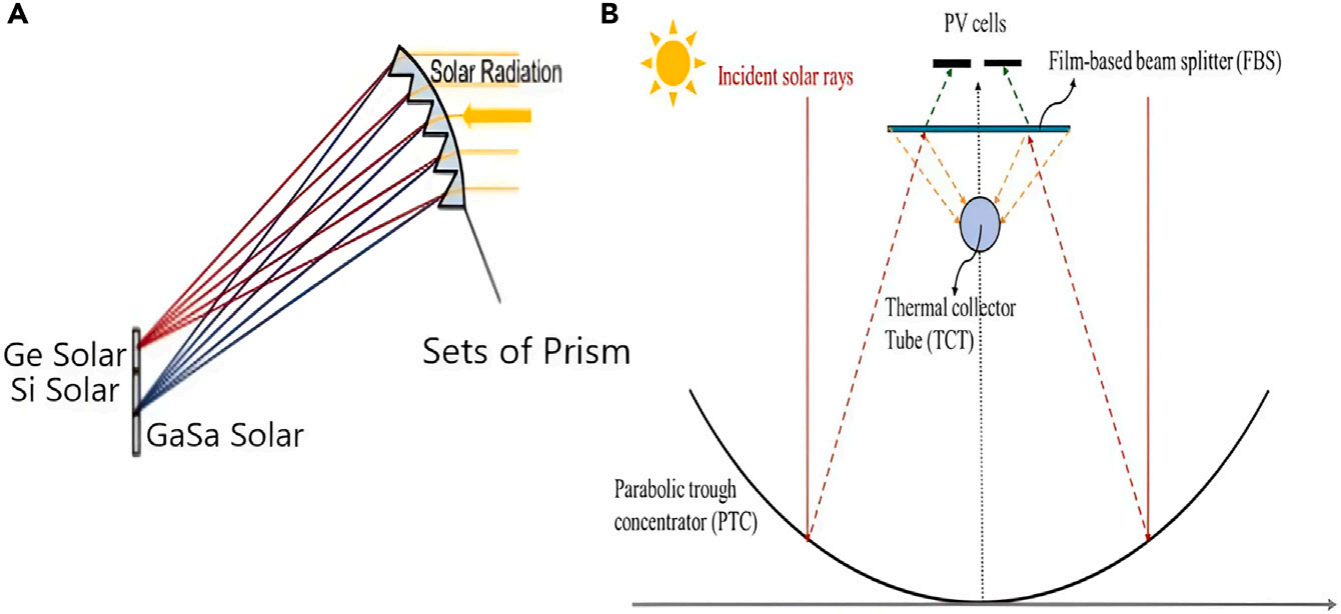
Recent advancement in intergratigting SBS with concnterated solar collector (A) A concentrating PVS utilizing prisms for spectral separation. This setup achieved concentration levels of 100x for monochromatic light and 17.5x for polychromatic light (ranging from 730 nm to 1000 nm). Reproduced with permission^48^ 2012 Optical Society of America. (B) PV/T system using both parabolic trough concentrator (PTC) and film-based beam splitter (FBS). Reproduced with permission^49^ Copyright 2023 Elsevier Ltd.

Another approach to reducing optical losses involves concentrating and splitting light in a single stage using a single device. This method minimizes the number of interfaces and thus reduces reflection losses. Such a device, illustrated in Figure 11, can be constructed from a set of prisms mounted on a curved surface^48^ or using dichroic concentrating mirrors, achieving both spectral splitting and concentration effects simultaneously. (Figure 11A) or using parabolic trough concentrator (PTC) and film-based beam splitter (FBS) (Figure 11B),^49^ achieving both spectral splitting and concentration effects simultaneously.

## Waste heat recovery

Apart from previously outlined technologies for improving the performance of SC, inventive approaches to reduce SC temperatures involve integrating a thermal absorber termed PV thermal (PVT).^50^ This entails augmenting a conventional PVS with a thermal absorber mechanism that channels excess heat to a working fluid, usable as thermal energy, showed in Figure 2C. The incorporation of a thermal absorber into a PVS can potentially enhance power generation in comparison to conventional PVS.^51^ Increased PVS temperatures amplify the fill factor of currents and reduce the voltage in the open circuit, resulting in an electrical performance decline.^52^ Consequently, incorporating thermal absorbers is vital to avert such a decline in performance. This study aims to scrutinize review papers on PVT technology, organizing them according to the methodologies utilized to establish a framework for forthcoming studies. This analysis will emphasize five critical aspects of PVT advancement: the use of polymer materials, PCMs, nano fluids, modification of absorbers, and mini/microchannel, allowing a comparison of the achievements of each method. Given the centrality of thermal conversion phenomena in this discourse, pertinent articles on STC, functioning akin to PVT absorbers, are also incorporated.

### Basics of PVT

#### Principle behind PVT technology

**Figure 12:**
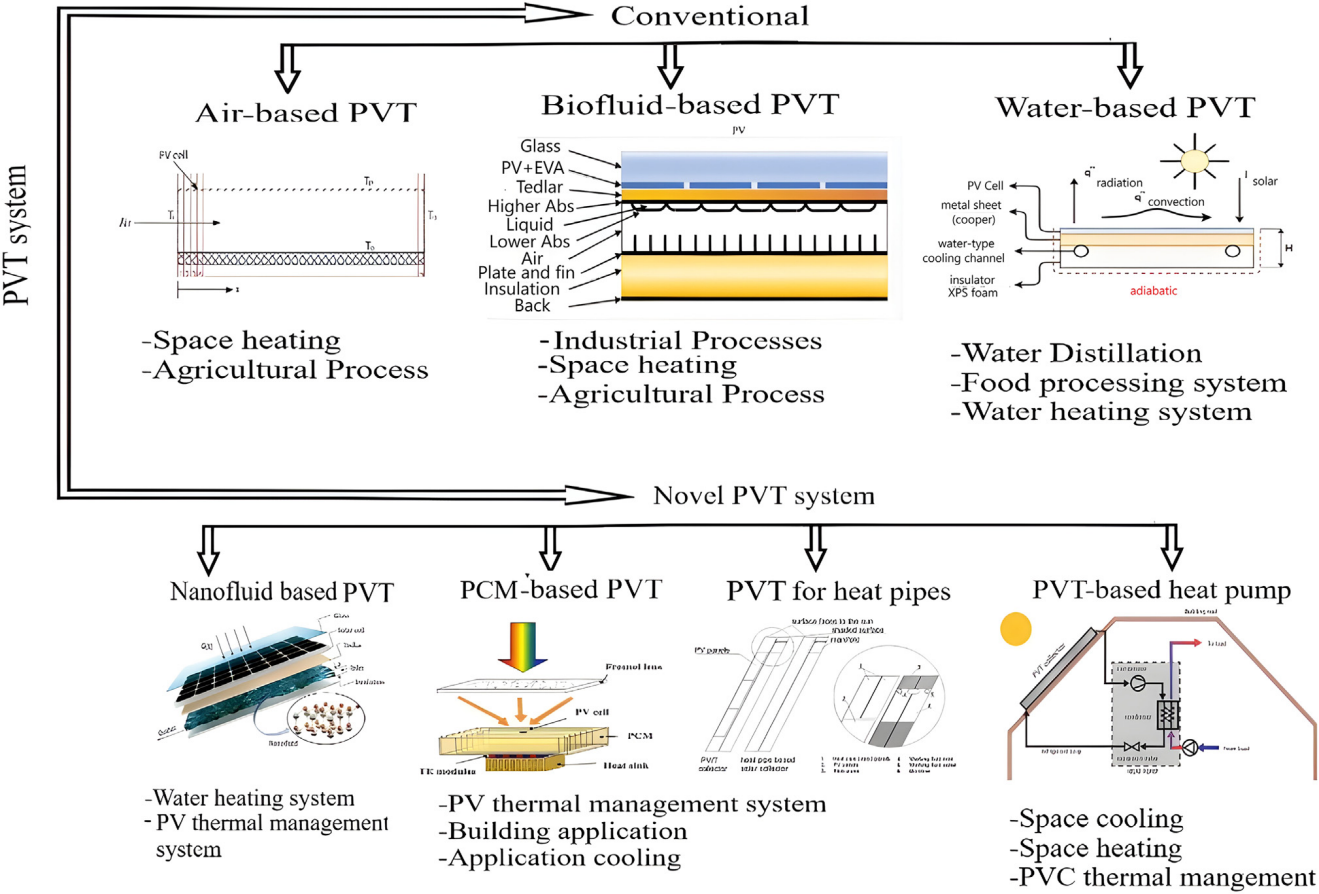
Conventional PVT and novel PVT systems (Comparison) Reproduced with permission^56^ Copyright 2014 Elsevier Ltd. Reproduced with permission^57^ Copyright 2021 MDPI. Reproduced with permission^58^ Copyright 2022 MDPI. Reproduced with permission^59^ Copyright 2022 MDPI. Reproduced with permission^60^ Copyright 2016 Elsevier Ltd. Reproduced with permission^61^ Copyright 2016 Elsevier Ltd. Reproduced with permission^62^ Copyright 2023 Elsevier Ltd.

As previously mentioned, PVT system is designed to harness surplus heat emitted by SC and address problems related to excessive heat that can degrade PVS’s performance (electrical). Advancements and exploration in PVT technology have led to various tailored solutions to satisfy market needs and enhance effectiveness. Noro et al.^53^ categorized the PVT system according to the heat extraction medium utilized: heat pipes, air, fluid, PCMs, and system using thermoelectric. They observed that PVT collectors, which are liquid-based, demonstrate higher thermal performance, as they promote a more even distribution of temperature due to the superior *h*_*m*_ and heat capacity in comparison to gases. Oh et al.^54^ further elaborated on a broader classification, encompassing various systems categorized by heat extraction medium, extraction methodology, system arrangement, solar input, and the utilization of spectrum filters.^55^ Moreover, Figure 12 ^56^^,^^57^^,^^58^^,^^59^^,^^60^^,^^61^^,^^62^ illustrates traditional and innovative PVT system methodologies and their integration into various application domains.^45^^,^^46^^,^^47^^,^^48^^,^^49^^,^^50^^,^^51^^,^^52^^,^^53^^,^^54^^,^^55^^,^^56^^,^^57^^,^^58^^,^^59^^,^^60^^,^^61^^,^^62^ Numerous factors, referred to as primary performance indicators, have the potential to impact PV efficiency. Various elements might influence these metrics, including environmental conditions, cable malfunctions, shading, interconnections, and wiring.^63^ Nonetheless, the criteria commonly employed for assessing PVT efficiency comprise SC temperature, thermal efficiency, electrical efficiency, and overall performance.^64^^,^^65^ These factors are affected by solar radiation, the *m*_*f*_ of the HT medium, and the accumulation of particulate matter. Considering PVS is deployed outdoors, external circumstances, particularly solar radiation levels, are paramount.^66^ Increased solar radiation intensity can reduce overall system effectiveness.^67^ The efficiency of PVS may deteriorate due to dirt buildup if regular cleaning is neglected.^68^ Controlling the θ can help mitigate dust accumulation. However, determining the optimal tilt angle is based on the specific installation site to maximize SE absorption while concurrently minimizing dust buildup.^69^ Hence, a meticulous balance between tilt angle adjustments and dust management strategies is imperative for optimizing SE absorption and mitigating dust buildup.

#### Factors influencing PVT performance

The heat absorption mechanism holds pivotal significance in PVT setups, as heightened temperatures within SC result in amplified current flow, consequently leading to enhanced power output. Nevertheless, PV systems are bound by particular temperature constraints. Augmented sunlight irradiance raises the outlet temperature by intensifying the heat exchange between SC and the circulating fluid inside the absorber, thereby augmenting thermal energy.^70^ In their experimental findings, Hassan et al.^71^ noted that an upsurge in solar radiation elevates PV cell’s temperature, consequently diminishing electrical efficiency. The following equations can mathematically describe the temperature dependency of PV performance.^72^^,^^73^

(Equation 1) $$P_{T_{c}}=\eta_{s}P_{in}k_{f}\left[1+\alpha \left(T_{c}-25\right)\right]$$

(Equation 2) $$\eta_{T}=\eta_{ref}-\mu \left(T_{c}-T_{ref}\right)$$

P_Tc_ is output energy (W)

η_s_ = efficiency in energy conversion at STC

*k*_*f*_*=* correction coefficient other than temperature.

P_in_ = irradiation (W/m^3^)

α = coefficient (correction) (rad/s^2^)

T_c_ = temperature of the module (°K)

η_T_ = Thermal efficiency of PV

η_*reff*_ = efficiency of a cell measured as reference temperature (T_ref_; °K)

μ = cell’s overall coefficient. (STC at 298 K)

The maximum power output of a PV module is

(Equation 3) $$P_{\max}=I_{max}\times V_{\max}$$

*I*_*max*_ = the maximum allowable operating current, and *V*_*max*_ = allowable voltage for power.

(Equation 4) $$\eta_{e}=\frac{P_{\max}}{I_{s}A_{panel}}$$

*I*_*S*_ = solar irradiation, and *A*_*panel*_ = PV panel surface area.

The thermal efficacy expression for the PVT system is as follows.

(Equation 5) $$\eta_{s}=\frac{Q_{u}}{I_{s}A_{c}}$$

*A*_*c*_ = surface area of thermal absorption, and *Q*_*u*_ = collection of useful heat.

(Equation 6) $$Q_{u}=c_{p}\left(T_{f}-T_{i}\right)$$

Where *m*_*f*_ = mass flow rate of the fluid entering the closed-loop system, *c*_*p*_ = specific heat capacity of the fluid, *T*_*f*_ = fluid’s temperatures at the system’s outlet, *T*_*i*_ = fluid’s temperatures at the system’s inlet, and *R* = universal gas constant.

During daily operations, the power generation steadily rises, reaching its peak around midday due to the increasing solar radiation, then gradually declines.^74^ Following 1 p.m., although solar radiation diminishes, the ambient temperature typically continues to climb, leading to performance deterioration.^75^ While it’s impossible to eliminate this decline, it can be alleviated by optimizing heat dissipation from the SC. In their study, Gholampour et al.^76^ conducted experiments on unglazed transpired STC. They found that the airflow rate has a notable effect on cooling efficiency. Higher airflow rates were related to enhanced efficiency, particularly evident when compared to the electrical-to-thermal ratio, which ranged from 1 to 4.

Haddad et al.^77^ documented analogous outcomes in their experimentation of PVT hybrid systems (water-cooled), highlighting how thermal efficiency is influenced by coolant volume. Following the principles of the second law of thermodynamics, minimizing exergy loss involves reducing EG. Leong et al.^78^ observed that increasing the wt. % of nanofluids and employing minimal *m*_*f*_ can effectively decrease EG. Furthermore, they discovered that TiO_2_ nanofluids exhibit lower total dimensionless EG than Al_2_O_3_, owing to TiO_2_’s superior *kp*, which facilitates enhanced HT. In a separate investigation, Marulasiddeshi et al.^79^ compared hybrid nanofluids (Al_2_O_3_–CuO) with standalone Al_2_O_3_ nanofluids, demonstrating that hybrid nanofluids attained lower EG values across numerous temperatures when contrasted with Al_2_O_3_ alone. Additionally, Liu et al.^80^ conducted an analysis on PVS with and without needle fin arrangements within the absorber tube, concluding that integrating needle fins resulted in a significant increase in EG, ranging from 49% to 54% compared to the alternative configuration, primarily due to heightened mixing creating velocity difference.

The thermal efficiency of PVT systems is not solely dependent on operative parameters. Still, it is also intricately linked to the absorber design, which is pivotal in the heat dissipation of SC.^81^ The central focus is heat dissipation, particularly concerning the absorber surface in contact with the cooling medium. Consequently, the size and absorber configuration emerge as crucial determinants. Bahrehmand and Ameri^82^ computationally compared STC with either a single-glass cover or a dual-glass cover featuring various sheet layouts of tin. The results indicated that extending the length of the channel to 4 m yielded maximum efficiency.

Ibrahim et al.^83^ compared the effect of axial flow and spiral flow in a rectangular channel generating hot water. Results showed that spiral flow power output compared to axial flow increased by 2.9 W. In a separate investigation into heat dissipation mechanisms, Ghale et al.^84^ scrutinized the efficacy in a rectangular microchannel with and without rib structure. Incorporating a rib increases HT contact surface, intensifies turbulence, and enhances HT capabilities; alterations in flow direction and increased cross-velocity further amplified HX. Nevertheless, modifications to the dimensions of the rib yielded varying outcomes. In contrast, a 28% increase in rib width augmented HT by 31%, and a 66% elevation in height translated to only a 15% enhancement in HT.

## Advancements in thermal management and absorption techniques to enhance the overall efficiency of PVT systems

In response to performance shortcomings, significant research efforts have focused on elevating the overall effectiveness of PVT through structural alterations.^85^ These approaches encompass restyling the absorber, incorporating mini/microchannels, adopting polymer materials, employing PCMs, employing working fluids as nanofluids, minimizing heat dissipation, and deploying augmentation mechanisms.^86^

### Design of absorber

As previously mentioned, the absorber is pivotal in tackling overheating concerns within PVS. Its geometric configurations considerably affect the heat dissipated from SC. Vengadesan and Senthil^87^ observe that several investigations strive to augment the HT surface area, instigate fluid turbulence, and prolong the duration of fluid distribution within the absorber. Nonetheless, these modification endeavors frequently yield adverse effects, such as heightened *Δp* or increased *f* losses, presenting challenges for upcoming research endeavors. Table 1 summarizes critical studies examining PVT with SC types.^88^^,^^89^^,^^90^^,^^91^^,^^92^^,^^93^^,^^94^^,^^95^^,^^96^

**Table 1:** Important research on PVT (hybrid systems) with SC type

Authors & Study type	SC used	Efficiencies (%)		Operating parameter
		Electrical	Thermal	
Ibrahim et al.^88^ Experimental	Poly-crystalline silicon	45	10	I_s_ of 800 W/m^2^ Ambient temperature of 25°C
Dupeyrat et al.^89^ Theoretical & Experimental	Mono-crystalline silicon	79	8.8	• Total radiation of 960 W/m^2^ • Differences between ambient and sky temperature are 30 and 33° • *v* of wind 3 m/s
Buonomano et al.^90^ Theoretical	InGaP/InGaAs/Ge triple junction	27–58	18–24	• Inlet fluid temperature of 150°C • Ambient and sky temperature of 26°C • *v* of wind is 5 m/s • Radiation of beam is 800 W/m^2^
Xu et al.^91^ Experimental	InGaP/InGaAs/Ge triple junction	54	28	• Ambient temperature ranges between 15°C and 17°C • Beam radiation of 700 W/m^2^
Calise and Vanoli^92^ Theoretical	InGaP/InGaAs/Ge	56–63	20–23	• Temperature of inlet water is 70°C • Temperature of ambient and sky is 25°C • Wind *v* of 5 m/s • Total radiation is 1000 W/m^2,^ and beam radiation is 800 W/m^2^
Cui et al.^93^ Theoretical	GaInP/InGaAs/G, c-Si, CIGS and GaAs			Ambient temperature of 27°C • Water inlet temperature of 20°C • SC temperature between 21°C and 35°C • *I*_*s*_ of 1000 W/m^2^
Sakellariou & Axaopoulos^94^ Theoretical & Experimental	Poly-crystalline silicon	69.68	18.91	• *v* between 0 m/s and 6 m/s • Ambient temperature between 24°C and 28°C
Karimi et al.^95^ Experimental	Mono-crystalline	43.6	14.5	• *I*_*s*_ of 920 W/m^2^ • Temperature of the SC is 20°C
Widyolar et al.^96^ Theoretical & Experimental	GaAs	37	8	• Beam radiation of 700 W/m^2^ • Fluid inlet temperature between 100°C and 500°C

Koech et al.^97^ examined various elements influencing the effectiveness of PVT air STC. Their findings revealed that, when maintaining a consistent number of PV cells, thermal efficacy experiences an upsurge while electrical efficiency undergoes a decline with the elongation of the collector length. This shift is ascribed to the heightened absorption of solar radiation across the inter-SC spaces within the PVT. Extending the collector’s length contributed to a reduction in heat dissipation, thereby optimizing the efficiency. Furthermore, they detected that adjusting Tedlar’s *kp* to 0.1 W/m·K positively impacted the system performance (electrical/thermal) by facilitating more efficient heat dissipation. Ibrahim et al.^88^ examined 7 distinct absorber configurations. They concluded that the design generating spiral flow attained the highest efficiency of 68% and the serpentine layout minimum of 45% with the highest outlet temperature of 31°C. The close tube layout demonstrated superior heat dissipation among different designs, yielding higher PV efficiency. Ali et al.^98^ investigated serpentine absorber channels in a PVT system employing an Al_2_O_3_–Cu/water nanofluid flowing within it. Their results indicated that a dual-serpentine channel augmented the hm more efficiently than a single serpentine. Furthermore, the electrical efficiency of the dual serpentine was observed to surpass that of the single serpentine by 2.37% across varying Re and nanofluid concentrations.

**Figure 13:**
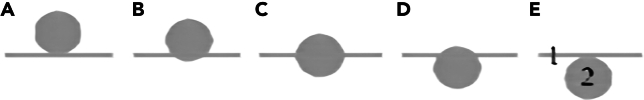
Different absorber and riser configuration used for evaluation 1. Absorber, 2. Riser, (A and B) Riser connected to upper surface, (C) riser connected to middle, (D and E) riser connected below. Reproduced with permission^99^ Copyright 2014 Avestia Publishing.

**Figure 14:**
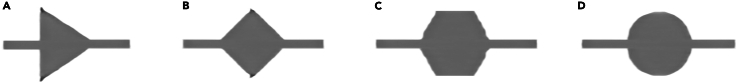
Different geometry of tubes used during evaluation (A) triangular, (B) square, (C) hexagonal, (D) circular. Reproduced with permission^99^ Copyright 2014 Avestia Publishing.

Ekramian et al.^99^ numerically (CFD) examined the thermal efficacy of different configurations of absorber risers (five distinct locations), as delineated in Figure 13,^99^ with PVT subjected to constant *I*_*s*_ of 800 W/m^2^ uniformly distributed over the absorber plate. The model illustrated in Figure 13A attained the utmost thermal efficiency. Successive declines in efficiency were noted as the riser descended (from the upper to lower surface of the absorber). This decline was attributed to the arrangement depicted in Figure 13A, which boasted a larger absorption surface area than alternative arrangements. Furthermore, Figure 14 ^99^ presents four riser shapes—square, triangular, circular and hexagonal and their influence on the HT mechanism. The riser with a circular cross-section showcased the peak thermal efficiency, surpassing the hexagonal, triangular, and square risers by 6.6%, 38.4%, and 11.2%, respectively.

**Figure 15:**
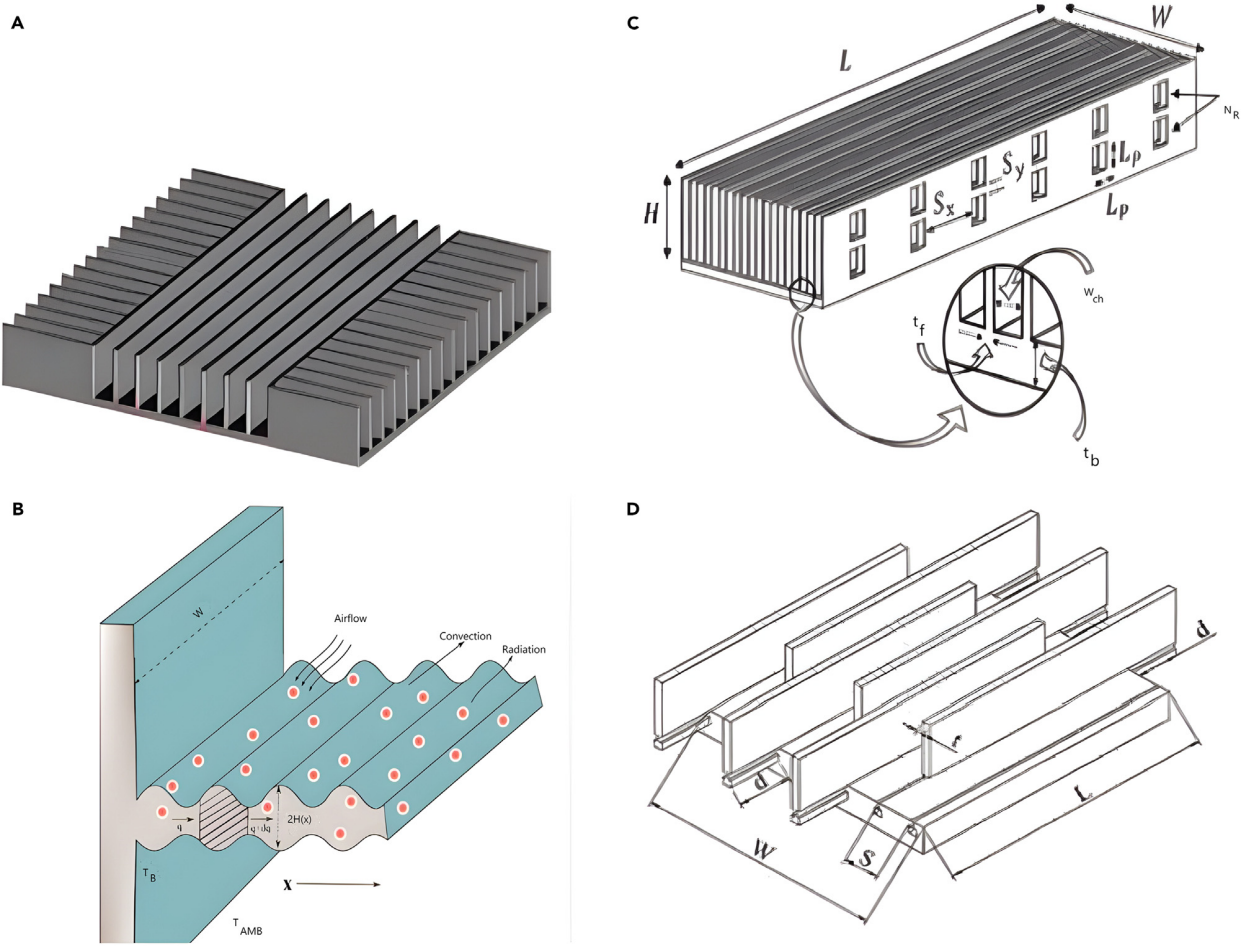
Different types of fin used by different researchers (A) Cross-fin heat sink. (B) Porous wavy fin (C) Laterally perforated-finned heat sinks. (D) Fin array. Reproduced with permission^101^ Copyright 2018 Elsevier Ltd. Reproduced with permission^102^ Copyright 2023 MDPI. Reproduced with permission^103^ Copyright 2017 Elsevier Ltd. Reproduced with permission^104^ Copyright 2020 Elsevier Ltd.

Elsafi and Gandhidasan^100^ theoretically and experimentally evaluated different fin configurations in a duel-pass FPC with air as a working fluid. Three different fin configurations were tested for electrical efficiency: triangle, rectangular, and parabolic, as well as diverse fin materials, such as steel, Al, Cu, Brass, and nickel. The simulation outcomes revealed that utilizing pin fins made of copper yielded superior performance compared to designs employing straight fins. A few examples of HT augmenting in PVC using novel fins are shown in Figures 15,^101^^,^^102^^,^^103^^,^^104^ 16 ^105^^,^^106^^,^^107^ and 17.^108^^,^^109^^,^^110^

**Figure 16:**
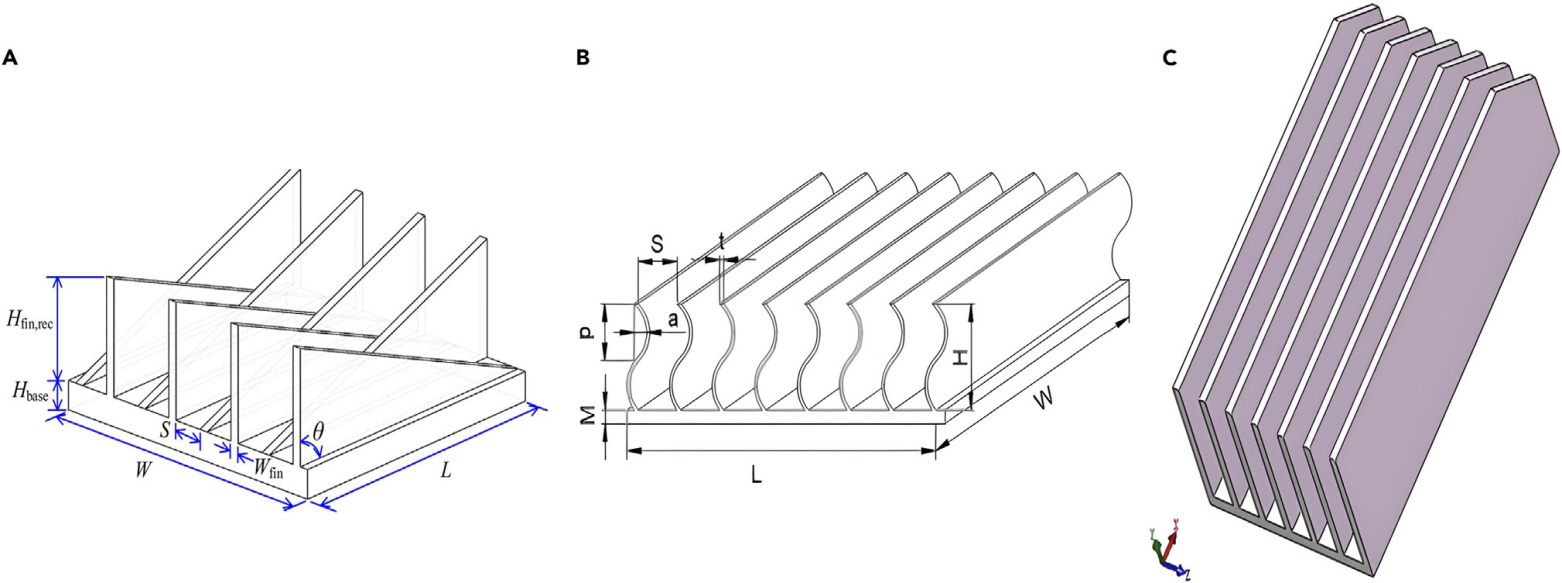
Different types of fin used by different researchers (A) Triangular fin. (B) Sinusoidal wavy fin. (C) Cut corner heat sink. Reproduced with permission^105^ Copyright 2019 Elsevier Ltd. Reproduced with permission^106^ Copyright 2019 Elsevier Ltd. Reproduced with permission^107^ Copyright 2018 Elsevier Ltd.

**Figure 17:**
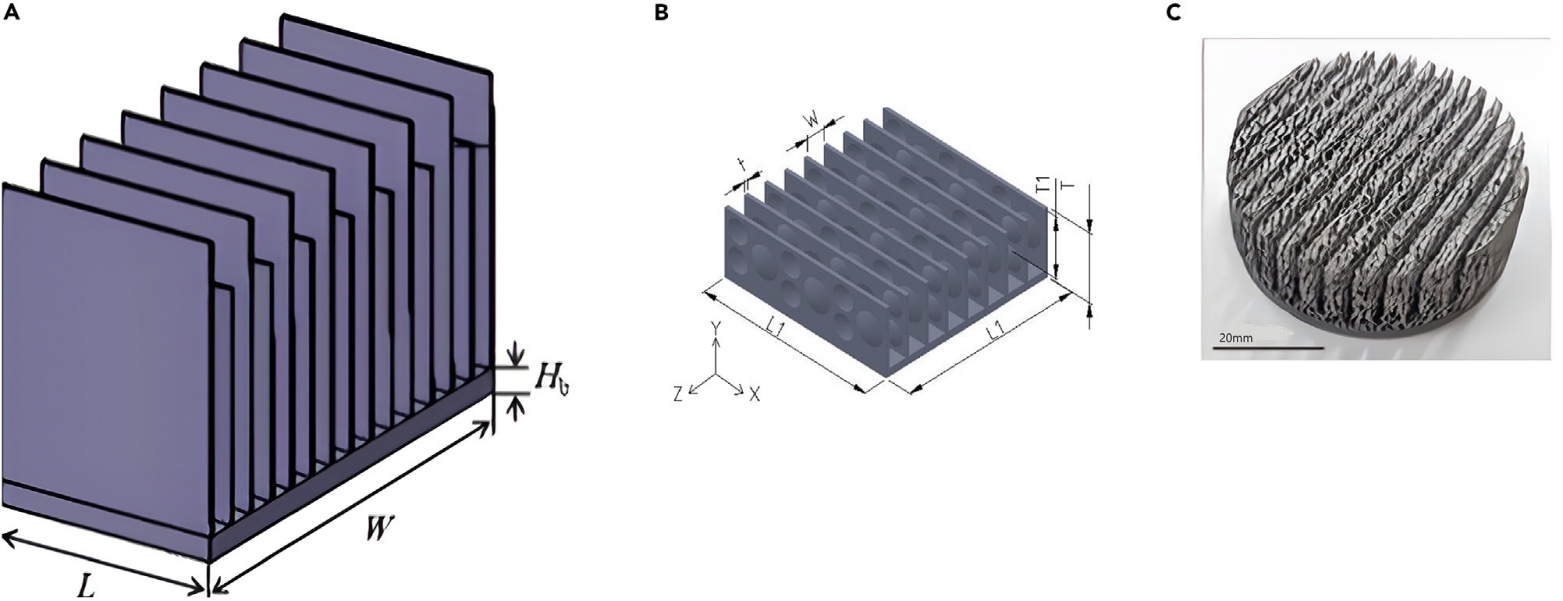
Different types of fin used by different researchers (A) Duel hight fin. (B) Fin array with alternate convex/concave dimples. (C) Aluminium foam-fin heat sink. Reproduced with permission^108^ Copyright 2017 Elsevier Ltd. Reproduced with permission^109^ Copyright 2020 MDPI. Reproduced with permission^110^ Copyright 2022 Elsevier Ltd.

Kazem et al.^111^ numerically examined water-cooling PVT systems with different configurations of flow channels (web type, direct type, and spiral type) with a constant *m*_*f*_ of 40 kg/h. The result indicates that SC temperature, on average reduced by 3°C, with the spiral flow (PVT system) shows maximum voltage, current and power of 17.7 V, 2.89 A and 51.3 W, respectively, reaching an overall efficiency of 35.0% in comparison to conventional axial flow type PVT design.

Missirlis et al.^112^ numerically examined the effect of three different manifold orientations at the inlet and outlet pipe within a polymer STC featuring a honeycomb framework with the blackened-absorbing medium. The most effective configuration to minimize heat loss is aligning the pipes with the honeycomb collector. This arrangement promoted linear fluid flow, leading to a uniform temperature in the collector.

### Micro/minichannels

**Figure 18:**
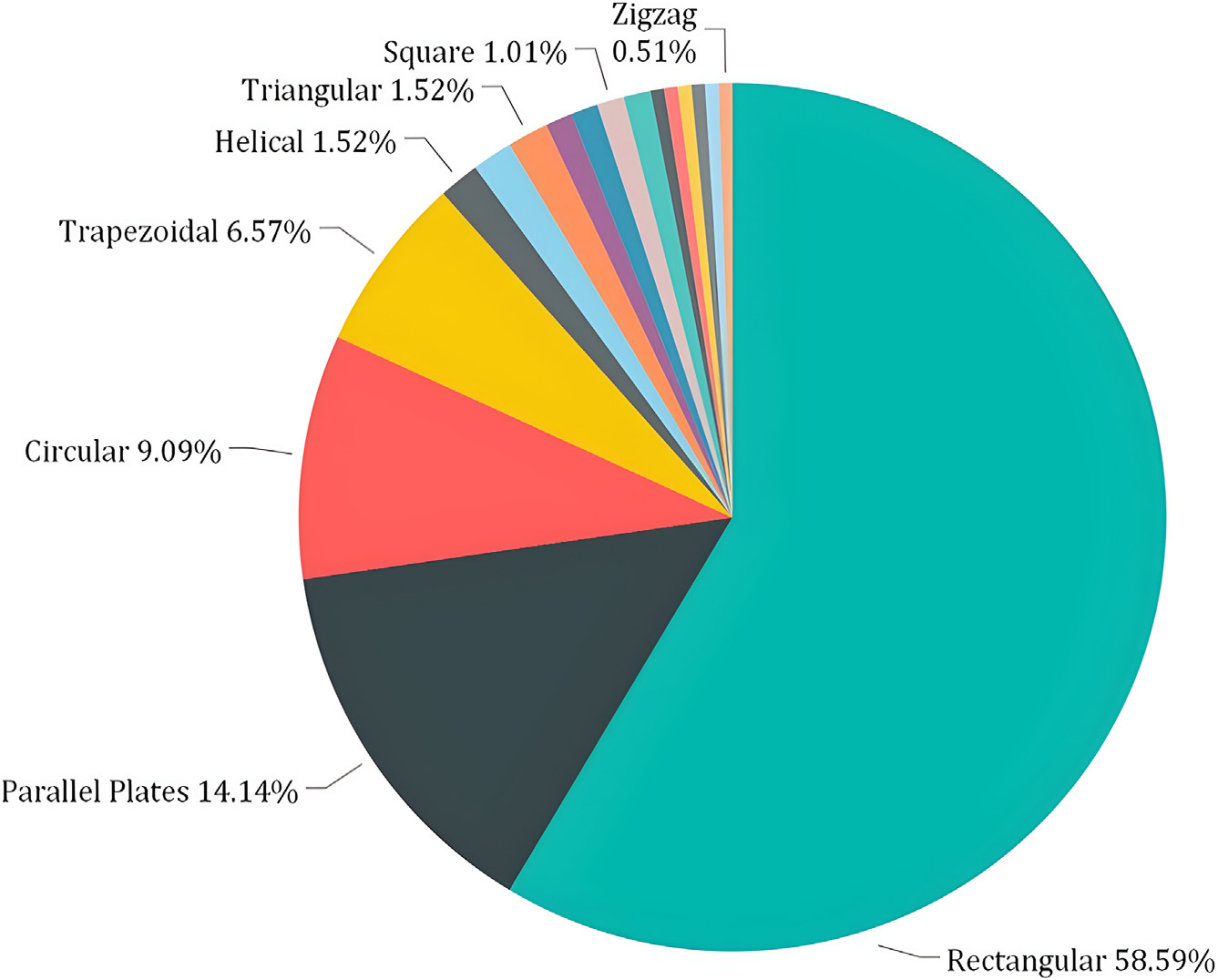
Developed in different geometries of mini/microchannel Reproduced with permission^117^ Copyright 2018 Elsevier Ltd.

As previously explored, the thermal collector within the PVT setup is pivotal in facilitating heat exchange. Microchannels have gradually evolved into the preeminent research arena. The size of the conduits within this arrangement bears notable significance; larger dimensions often correlate with diminished efficiency.^113^^,^^114^ Hence, opting for smaller diameters becomes imperative to optimize equipment performance. Tuckerman and Harmony^115^ proposed that employing liquid-cooled HX with minute dimensions could improve the heat transfer mechanism, sparking a global trend in microchannel technology. Numerous investigations have been undertaken to incorporate mini/microchannels into PVT, thus driving renewable energy.^116^ Figure 18 ^117^ illustrates various geometrical configurations of mini/microchannels developed. Table 2 overviews significant research endeavors concerning mini/microchannels.^118^^,^^119^^,^^120^^,^^121^^,^^122^^,^^123^^,^^124^^,^^125^^,^^126^^,^^127^

**Table 2:** Summary on remodeled absorbers of PVT systems

Authors Type of Study	Types	Efficiencies (%)		Conclusions
		Electrical	Thermal	
Deng et al.^118^ Experimental.	L-shaped dual micro heat pipe arrays		61.48% at 200 m^3^/h	Heat collection efficiency rises with a rise in air flow rate.
Shahsavar et al.^119^ Experimental.	Plain, staggered, and parallel grooved helical microchannel		53.6	Staggered grooves perform better than others in both energetic and exergetic aspects.
Robles et al.^120^ Numerical	Aluminum microchannel attached to absorber plate in water heater (solar)		13	Improved design leads to high thermal efficiency.
Sharma and Diaz^121^ Numerical	U-shaped flat tube absorber with microchannel		20.7 at 192.9°C	Minichannels offer superior performance, but operational conditions must be taken into account.
Radwan et al.^122^ Numerical	Microchannels as heat sink in low-concentration PV	18.5	62.5	Microchannel provides effective cooling, reducing SC temperature.
Oyinlola et al.^123^ Experimental and theoretical	16 microchannels measuring 0.5 × 2 × 270 mm			*Re* range of 10–100. Adding insulation on both sides of the plate minimizes heat loss.
Peng et al.^124^ Theoretical	Geometry optimization based on meteorological data and sensitivity analysis.			Energy collection improved by 6.57%–16.37%
Varghese et al.^125^ Numerical	serpentine microchannels in MEMS			*Re* between 50 and 1000 HT rises due to a surge in HT surface area and secondary flow.
Tomar et al.^126^ Experimental & Theoretical	This study used a PV-HPCW heat pump system with a wickless heat pipe integrated aluminum veneer curtain wall as a solar thermal collector system.	9.44	20.22	The new system shows proper resource consumption and compact size.
Brahim and Jemni^127^ Experimental & Theoretical	Acetone wickless heat pipe solar panel PVT system is investigated using heat pipe number, collector surface area, wind velocity, water inlet temperature, θ and inner heat pipe behavior.	12.52	43.75	Overall efficiency of the PVT/WHP module was 56.27%.

Wang et al.^118^ introduced L-shaped dual micro heat pipe arrays in FPC to enhance the thermal efficiency of solar air collectors by minimizing heat loss and optimizing HT from the evaporation section. The collector demonstrates promising performance with average heat collection efficiencies of 49.72%, 55.69%, 59.37%, and 61.48% for air *m*_*f*_ of 80, 120, 160, and 200 m^3^/h, respectively. Under the highest airflow rate (200 m^3^/h), the temperature in the evaporation section reduces to 25.28°C.

Shahsavar et al.^119^ examined three designs for microchannels. When paired with a nanofluid PVT, they observed that a staggered layout performed better than parallel and conventional designs. In addition to selecting nanofluid to boost thermal efficacy, the staggered layout’s arrangement showed enhanced mixing of fluids. Peak thermal outputs of 33.6, 37, and 29.1 W were noted for parallel, staggered, and conventional arrangement at a *m*_*f*_ of 80 kg/h with NP concentration of 2.0%.

Robles et al.^120^ Experimentally investigated a microchannel design connected to headers instead of flat fins to HT to the fluid, and its performance was compared with that of a traditional copper FPC under identical conditions. The aluminum-based mini-channel solar water heater improved efficiency through an enhanced thermal design. Results indicate an average 13% increase in thermal efficiency with the mini-channel collector and improved daytime energy collection. A mathematical model of the mini-channel collector is developed and validated using experimental data collected year-round. The model demonstrates that these collectors maintain performance despite aluminum’s lower *k*_*p*_.

Sharma and Diaz^121^ scrutinized the repercussions of *m*_*f*_ at diverse inlet temperatures and deduced that augmenting the *m*_*f*_ amplifies efficiency. Raising the temperature at the inlet at different *m*_*f*_ led to an efficiency downturn. Upon comparison with earlier work, the current design outperformed. This enhancement was ascribed to the mini channel’s expanded HT region, fostering efficiency across all scenarios. Analogous to other investigations, they noted a heightened *Δp* with an escalating *m*_*f*_.

Radwan et al.^122^ numerically studied, a novel cooling approach using a microchannel heat sink is proposed for low-concentration PV systems. This research explores how various operational parameters like concentration ratio, cooling flow rate, wind speed, ambient temperature, and inlet temperature of the cooling liquid impact the performance of the LCPV thermal system. The findings suggest that employing a microchannel heat sink is highly effective, particularly for concentrated PVS. This leads to a significant decrease in SC temperatures and achieving uniform temperature distribution. For instance, at a microchannel flow *Re* of 100, it is observed that at a concentration ratio of 20, local SC temperatures range between 33.5°C and 35.6°C. In contrast, at a concentration ratio of 40, temperatures vary from 37°C to 41°C. Additionally, at a concentration ratio of 40, electrical efficiency reaches 18.5%, with thermal efficiency peaking at 62.5%. The estimated power loss due to microchannel friction is approximately 0.4% of the electrical power output.

Oyinlola^123^ examined a temperature plot within an absorber plate showing sixty microchannels measuring (0.5 × 2 × 270 mm) that were 1 mm spaced (with a 3 mm pitch), under Re range 10–100 with fluid inlet temperatures ranging between 5°C and 60°C. Variations in temperature profiles between the plate and fluid diminished with increasing *m*_*f*_. While HT minimally affected the fluid’s temperature, it dropped the temperature gap between the plate and the fluid. Notable alterations in the axial *kp* study detected a 10% decline in the plate’s initial and final temperature.

Peng et al.^124^ experimentally investigated the importance of using microchannels in PVT evaporators to augment solar energy absorption based on regional changes. Subsequently, the geometries of the microchannel-PVT evaporator are optimized by integrating suction superheat requirements. The findings reveal that the fixed-size microchannel-PVT evaporator is unsuitable for different regions. The refrigerant’s superheat requirement effectively reflects the combined impact of solar radiation and ambient conditions. Optimal micro-channel diameters and lengths decrease with higher solar radiation and ambient temperatures, ranging from 1.6 to 3.2 mm and 1.67 to 3.76 m, respectively. Overall, the evaporator’s performance predominantly depends on the refrigerant *m*_*f*_, with an average sensitivity coefficient of 1.97.

Varghese et al.^125^ numerical investigated the effect of serpentine geometry of microchannels used in concentrated PVT with a concentration ratio of 20 at *Re* between 50 and 1000. Compared to straight microchannels, the MEMS heat sink serpentile microchannel shows lower thermal resistance, up to 64%, and a 126% higher pumping power. The result also indicated that increasing *Re* increases pumping power irrespective of geometric variation. Also, *Nu* and Poiseuille numbers show a higher value when compared to straight microchannels.

### Polymer materials

The examination of polymer substances, mainly in thermal absorption, is primarily motivated by lower production expenses, prompting scholars to explore substituting metals with other materials like polymers. Furthermore, based on IEA (Solar Heating and Cooling Program Task 39),^128^ polymeric materials were emphasized for PVT setups, providing advantages like protection against overheating and surface coatings, especially for collectors. Despite having advantages like lightweight and surface coatings in contrast to traditional glass, a specific problem of endurance exists with polymers.^129^ Particularly during outdoor installation, the endurance of polymer substances is prone to deteriorate with time. The degradation of polymer materials stems from numerous factors is depicted in Table 3.

**Table 3:** Degradation factors for polymer

Degradation^130^	Primary Factor
Thermal degradation^131^	Rise in Temperature
Oxidative degradation^132^	Temperature & water
Chemical degradation^133^	Use of chemicals
Biodegradation^134^	Enzymes from microorganism
Hydrolytic degradation^135^	Temperature & water
Mechanical degradation^136^	Stress(Mechanical)
Photo-oxidative degradation^137^	Oxygen & UV radiation

Chow^136^ additionally indicated that polymers serving as absorber materials in this setup exhibit low *kp*, significant thermal expansion, and restricted operational temperature range. The classic *kp* range for polymer materials spans between 0.1 and 0.5 W/m·K, showcasing a considerable disparity with metal materials concerning heat absorption. The diminished capacity to transfer heat energy arises from the polymer chain morphology, which is often entangled and chaotic.^137^ Nonetheless, specific polymers categorized as thermally transparent polymers lack heat absorption capabilities, thus directly dissipating heat into ultra-cold space, resulting in efficient cooling effects.^138^ Furthermore, the flexibility in the choice of polymer presents avenues for researchers to mitigate these shortcomings. Table 4 provides an overview of research investigating the utilization of polymer materials in PVT systems.^139^^,^^140^^,^^141^^,^^142^^,^^143^^,^^144^^,^^145^^,^^146^^,^^147^

**Table 4:** Recent articles using polymer in PVT setup

Authors	Types	Efficiencies (%)		Conclusions
		Electrical	Thermal	
Erkata Yandri^139^ Experimental	PMMA I_o_ = 400–1000 W/m^2^*m*_*f*_ = 200–300 g/min	7.87	82.6	Efficiencies like electrical and thermal exhibit inverse reactions to rising solar temperatures
Filipovi’c et al.^140^ Experimental & Numerical	PVC and polyamide		76.79	The thermal efficiency demonstrated opposing trends in response to variations in the number of air gaps and the *kp* of the base material.
Wang et al.^141^	Poly(3-hexylthiophene)			Maximum output voltage of 58 mV under 1 kW m^−2^ solar irradiation
Nishit and Bekal^142^ Experimental	Al_2_O_3_+ DI water+ surfactant (Sodium dodecyl sulfate + Cetyltrimethyl ammonium bromide)		27.7	Applying Al_2_O_3_ improves system performance to offset the limitations inherent in polymers.
Ariyawiriyanan et al.^143^	absorber fabricated from polyethylene			Manufacturing costs reduce by 4 times.
Mintsa Do Ango et al.^144^	Polycarbonate (PC)			10 mm air gap gives the best efficiency.
Chen et al.^145^	PC			Aluminum STC surpasses their polymer counterparts in terms of thermal efficiency.
Kim et al.^146^ Experimental.	PC + CNT (˂ 15wt. %) constant *I*_*0*_ of 835 W/m^2^ .			Extending length does not affect the thermal efficiency of the absorber.
Nishit et al.^142^	polymer with Al_2_O_3_ NP		27.7%	
Yang et al.^147^ Experimental and numerical	Paraffin wax +ZnO/CuO			PCM-NP effectively maintain low surface temperature.

Yandri^139^ researched the heat energy generated by an electric current flowing through conductive materials within a PVS, employing a composite of PMMA with a copper sheet as the absorber. The absorber (made of composite) with no water circulation displayed a slower decline in electrical effectiveness with rising surface temperature, displaying its effectiveness in absorbing and retaining heat within the PMMA. Upon the introduction of water circulation through the absorber, the temperature of the collector was maintained within the range of 40°C–50°C. At constant radiation and a coolant *m*_*f*_ of 300 g/min, a maximum of 82.5% in thermal efficiency is recorded. Filipović et al.^140^ prepared a polymer-based STC incorporating polyvinyl chloride used in the main section and polyamide for water connections. Thermal efficiency during stagnation periods was evaluated. They investigated the impact of emission, air gap and *kp* on thermal efficiency. Raising the air gap caused a 2% improvement in efficiency at increased water temperatures. However, raising *kp* from 0.14 to 10 W/m·K caused a decline in efficiency, chiefly in scenarios with 10 mm and 30 mm air gaps.

Wang et al.^141^ numerically studied the consequence of organic PV materials in solar evaporators, which have extensive light absorption capability and efficient heat conversion. Poly(3-hexylthiophene), or P3HT, was used for solar-thermal conversion efficiency of 11.26%. This was embedded into non-woven fabric. Under simulated solar irradiation of 1 kW m^−2^, this device achieved an evaporation rate of 1.04 kg m^−2^ h^−1^ and generated an output voltage of 58 mV.

Nishit and Bekal^142^ highlighted the tendency for synthetic-based STC to perform below par compared to standard flat-plate solar panels. In response, they devised a synthetic PVT system employing Al_2_O_3_ nanofluid (as the principal operating liquid. To ensure nanofluid stability, they suggested incorporating surfactants at varying concentrations. This led to a significant instantaneous efficiency boost of approximately 27.7% when juxtaposed with synthetic STC. The superior thermal conductivity of Al_2_O_3_ over water contributes to enhanced heat absorption during solar exposure.

Mintsa et al.^144^ tried to increase the performance of FPC by employing polymers in place of absorber materials like copper/aluminum. They noted that elongating the length of collectors did not affect the system significantly because of the direct correlation between absorber length and the *m*_*f*_ of coolant. The heat-capturing capacity intensified upon increasing length, and increasing the *m*_*f*_ (coolant) improved heat dissipation. A few factors, like the thickness of the air gap, coolant *m*_*f*_, and coolant inlet temperature, affect the heat dissipation upsetting performance.

Chen et al.^145^ empirically studied FPC incorporating a multichannel absorber of honeycomb-shaped made from polymer materials. The modification in absorber geometry aimed to enhance HT regardless of polycarbonate’s inherently stumpy *kp*. Their results showed that conventional STC exceeded the proposed polymer STC thermal efficiency by 7–14%. This pattern remained consistent irrespective of changes in polymer materials.

Kim et al.^146^ altered the materials of the absorber to polycarbonate to streamline costs while augmenting HT by integrating fillers (CNT) into the polymer layer, significantly enhancing the STC’s thermal efficiency, Furthermore, redesigning the mounting component and increasing length reduced air entrapment, achieving higher efficiency.

Nishit et al.^142^ studied experimentally the effect on SWH using Al_2_O_3_ nanofluids of 0.05 vol %. The stability was increased by adding surfactants like sodium dodecyl sulfate (SDS) and cetyltrimethyl ammonium bromide. Results demonstrated that using Al_2_O_3_ nanofluid in the polymer solar collector raised the thermal efficiency by 27.7% compared to a polymer solar water heater using water as the heat-absorbing fluid.

Yang et al.^147^ experimentally and numerically studied how using phase change materials (PCM) in glass envelope devices enhances thermal regulation, boosting both comfort and energy efficiency in buildings. Adding NP presents promising applications for improving these systems’ solar energy collection and storage capabilities. The findings indicate that the presence of NP reduces the transmittance of the enhanced paraffin, with a slight increase occurring as temperature rises. The nanoparticles exhibit significant light attenuation, leading to a maximum scattering proportion of 6.3%. Enhanced *kp* is observed with 5.87% and 13.12% improvements at a *v*_*f*_ of 5 × 10^−4^ vol % for ZnO and CuO - NP, respectively. Further, Rahman et al.^148^ identified the potential of nanoparticles in PVT.

### PCM

**Figure 19:**
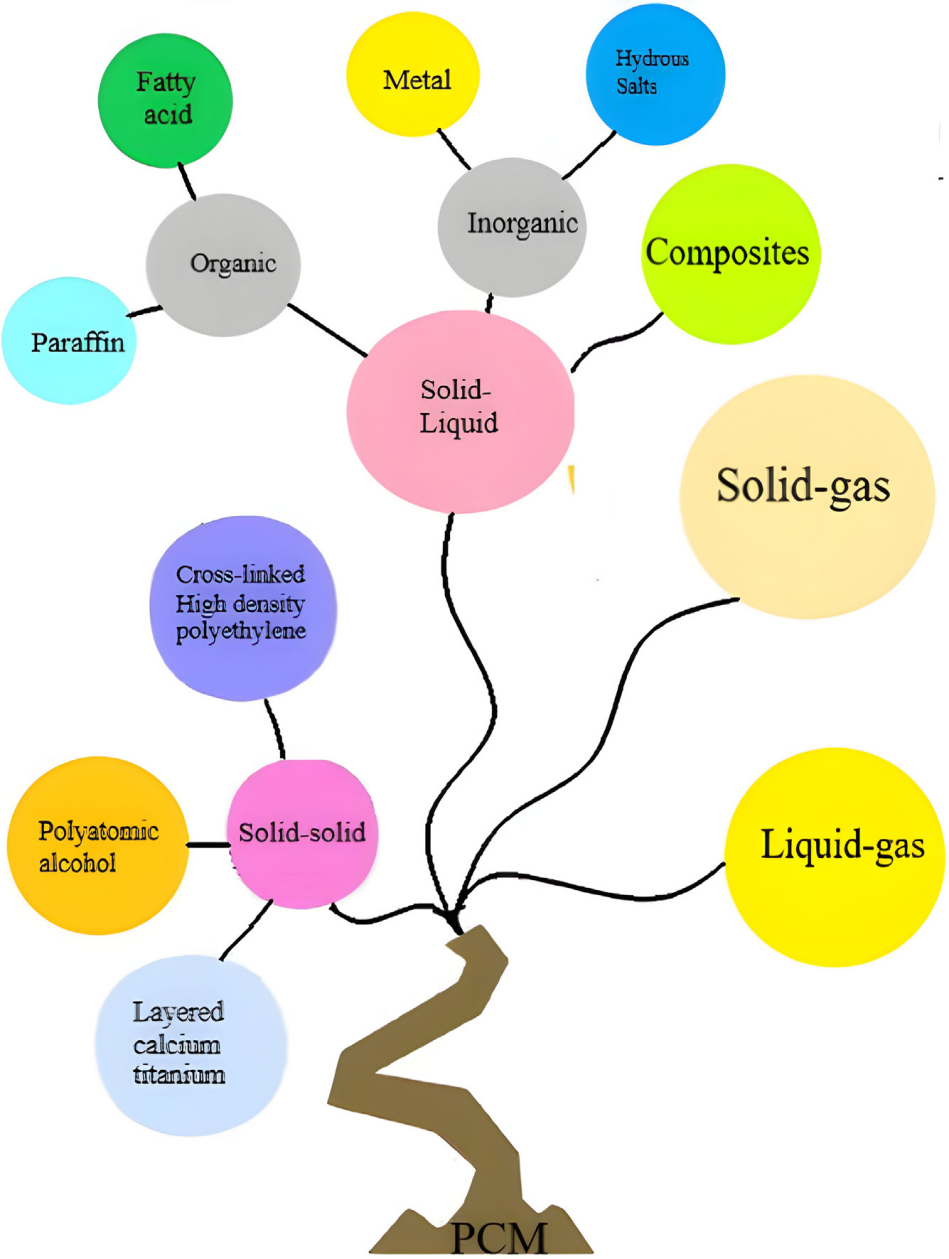
PCM Classification Reproduced with permission^150^ Copyright 2015 Elsevier Ltd.

PCMs were first acknowledged as effective LHTES substances by Telkes and Raymond^149^ during the 1940s. Generally, PCMs exhibit high efficiency in absorbing/releasing significant thermal energy under limited temperature ranges, rendering them appropriate for diverse applications, like solar energy collectors, WHR, and intelligent housing. Different PCM categories are illustrated in Figure 19.^150^

**Figure 20:**
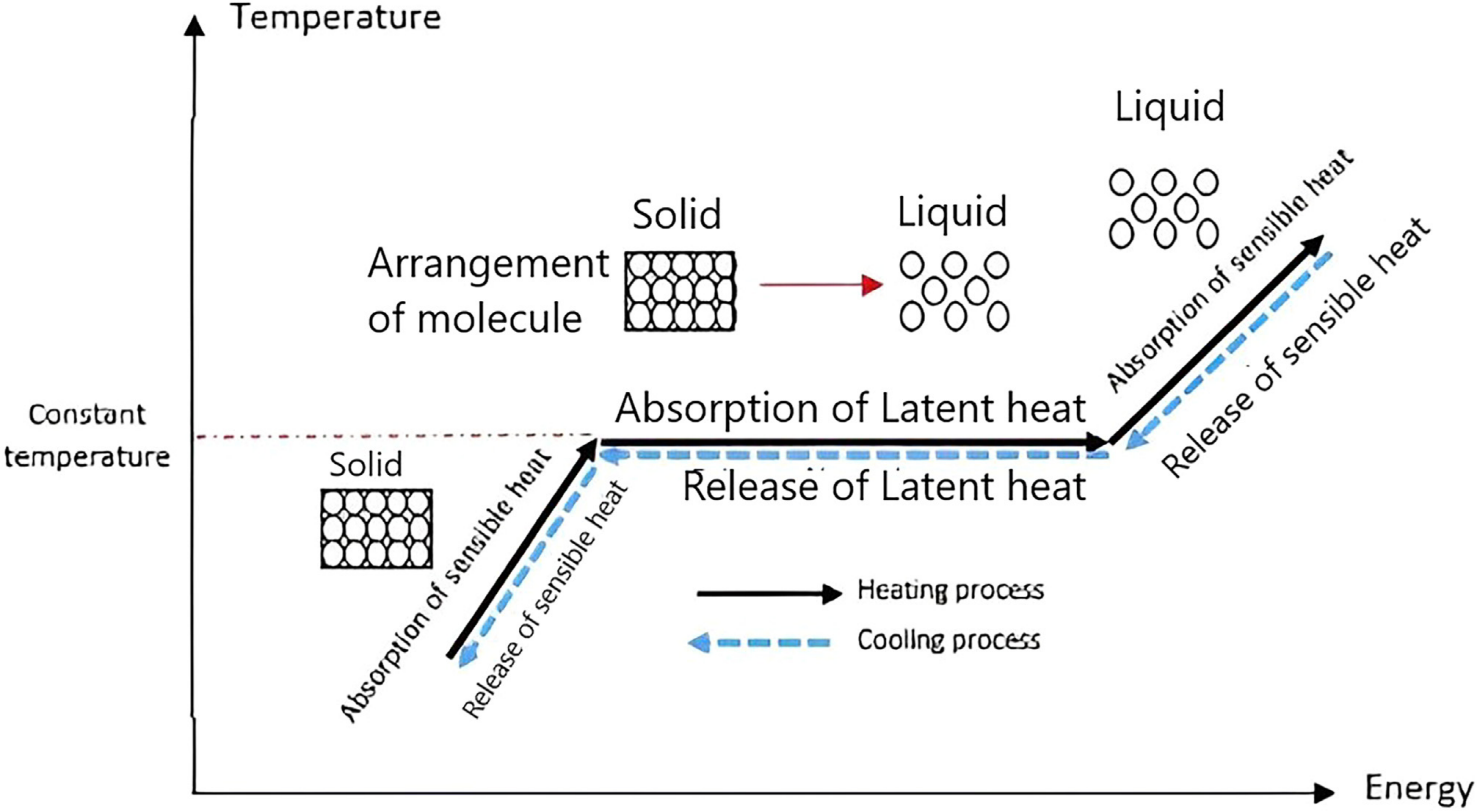
Thermal energy storage in PCM to rise temperature Reproduced with permission^151^ Copyright 2019 Elsevier Ltd.

PCMs captivate sensible heat till they attain their solidifying/melting point; after that, they absorb latent heat and continue undergoing a phase transition, as depicted in Figure 20.^151^

PCMs offer considerable potential for heat storage, as they can store 5 to 14 times more heat per unit volume compared to sensible heat storage materials. Their key benefit lies in their ability to absorb and release heat while maintaining nearly constant temperatures. Table 5 provides information on the melting temperatures, latent heat of fusion, and *k*_*p*_ of the commonly used PCMs.^152^^,^^153^

**Table 5:** Properties of few PCMs

PCM	Melting Temp, (°C)	Latent Heat, (kJ/kg)	*k*_*p*_, (W/(m·K))	
			Liquid State	Solid State
**Organic**				

C_14_H_30_	6	229	–	0.21
C_18_H_38_	28	244	0.14	0.35
C_8_H_16_O_2_	16	148.6	0.14	–
C_10_H_8_	80	147.7	0.13	0.34
C_4_H_10_O_4_	118	339.8	0.32	0.73

**Inorganic**				

CaCl_2_ 6 H_2_O	29	190.8	0.54	1.08
Ba(OH)_2_ 8 H_2_O	78	265.7	0.65	1.25
Mg(NO_3_)_2_ 6 H_2_O	89	162.8	0.49	0.61
MgCl_2_ 6 H_2_O	117	168.6	0.57	0.69

The PCMs obviate any flowing medium’s need to dissipate heat, thereby eliminating the need for external energy input, such as pumping power within PCM systems.^154^^,^^155^ Recently, researchers have directed their attention toward harnessing the distinctive heat dissipation properties of PCMs within PVT systems, as outlined in Table 6.^156^^,^^157^^,^^158^^,^^159^^,^^160^^,^^161^^,^^162^^,^^163^^,^^164^^,^^165^^,^^166^

**Table 6:** Summary of pieces of literature exploring PCM in PVT systems

Authors	Types	Efficiencies (%)		Conclusions
		Electrical (%)	Thermal (%)	
Imam et al.^156^ Experimental.	Thin PCM film under absorber plate	12.5	70.9	The incorporation of PCM in PVT systems at the absorber plate stores latent heat results in improved thermal performance after 5 p.m. to around 40%
Sardarabadi et al.^157^ Experimental	Paraffin wax + ZnO nanoparticle(0.2 wt. %)		48	The utilization of PCM surpasses the application of nanofluids in performance.
Yazdanifard et al.^158^ Numerical	RT25 or S27 with NP	14	46	PCM with a higher melting point enables accelerated HT, expediting system stabilization toward a steady state.
Madurai et al.^159^	HS 29 PCM			The performance of the PVT improved.
Manoj et al.^160^ Experimental	Nanocomposite PCM			*kp* of paraffin wax was increased by 22.78%
Diallo et al.^161^ Numerical		12.18	55.26	Elevating the water inlet temperature leads to heat dissipation.
Tyagi et al.^162^ Experimental	Hybrid cooling-MWCNT is the coolant, and PCM is the passive cooling.	12.81	1.66	Hybrid cooling of PVT improves performance.
Simón-Allué et al.^163^ Experimental	Compared glazed to unglazed, with aluminum or polymetric material.			PCM doesn’t notably alter thermal performance but enhances heat generation distribution.
Jurčević et al.^164^ Numerical.	organic PCM			Reduces the CS temperature
Bhutto et al.^165^ Experimental	Ag and Gr hybrid NP fatty acid PCM			LAG-3.5 nanocomposite augmented *kp* by 57%
Xu et al.^166^ Experimental.	Hexadecanol-palmitic acid/expanded graphite eutectic composite PCM			Output voltage and power of PV panel improves.

Imam et al.^156^ examined the impact of PCM on the outlet water temperature. During peak radiation hours, solar energy is captured by the PCM in the storage unit. This stored thermal energy is then utilized to heat the water during periods of lower radiation. A compound parabolic concentrator (CPC) was mounted on the upper surface of the thermal collector, and a thin layer of PCM was placed beneath the absorber plate to store the collected latent heat. The system maintained a consistent flow rate and achieved the maximum outlet water temperature across both years. Additionally, the PCM system exhibited a more gradual decline in solar efficiency under lower solar radiation than without PCM.

Sardarabadi et al.^157^ experimentally examine the impact of using a ZnO/water nanofluid with a PCM as a cooling agent in a PV collector system that utilizes fluid/nanofluid. Two similar PV thermal systems were designed and constructed for the experiment: one incorporating a PCM (PVT/PCM) and the other without it (PVT). The performance metrics, including surface temperature, thermal efficiency, and electrical efficiency, were measured and compared between the systems, and a conventional PV module was used as a reference. Additionally, the performance of the nanofluid was assessed against pure deionized water. The findings reveal that the average electrical output in the PCM/nanofluid collector system was enhanced by over 13% compared to the conventional PV module. Using nanofluid instead of deionized water resulted in a nearly 5% increase in average thermal output for the PVT system. When PCM was also used (i.e., in the PVT/PCM system), the thermal efficiency improved by almost 9% without additional energy consumption. According to exergy analysis, the combined use of nanofluid and PCM significantly boosts the overall exergy efficiency of the system by more than 23% compared to a traditional PV module.

Yazdanifard et al.^158^ numerically investigated PVT systems employing two distinct PCMs, namely RT25 or S27, with NP (Ag+water) to refine operational efficiency. Various placements of PCM layers, like the absorbent (optical) filter above and below, were tested. The findings highlighted the greater efficiency of S27 over RT25, attributed to its thermal properties crucial for efficient heat transfer. S27 showcased higher efficiency (electrical) compared to RT25 in solid state. Positioning PCM beneath the nanofluids caused a notable boost in energy efficiencies of 11%.

Manoj et al.^160^ studied an all-glass evacuated tube SWH’s TES capacity by incorporating nanocomposite PCM (NCPCM). The PCM used was paraffin wax, while the NCPCM consisted of paraffin wax combined with 1 wt % SiO_2_ NP. Experimental results were obtained from real-time measurements of the all-glass evacuated tube SWH, which featured built-in TES and operated using thermosyphonic flow. The experiments included 3 scenarios: no PCM, with PCM, and with NCPCM. The testing monitored the temperature variations in the tank water over 24 h the following day, with water wholly replaced every 12 h. The results indicated that the water temperature recorded at 6:00 a.m. the next day increased to 37°C using PCM and 39.6°C with NCPCM, compared to 33.1°C without PCM. Energy and exergy efficiencies were found to be 58.74%, 19.6% without PCM, 69.62%, 22.0% with PCM and 74.79%, 24.6% with NCPCM. Additionally, the *k*_*p*_ of paraffin wax was augmented by 22.78% by adding SiO_2_ NP.

In a related study, Diallo et al.^161^ conducted a computational analysis of a PVT system featuring an evaporator (with microchannel heat pipe) and condenser (with triple PCM-HX). The research examined how following factors: environmental variables, structural constraints, and HT fluid properties influence system’s efficiency. Upon comparing the performance of the Hybrid PVT with traditional PVT, the electrical efficiency improved at poor solar radiation, inferior ambient temperature, lower coolant inlet temperature, higher packing factor, and reduced coverage. Additionally, efficiencies like thermal and electrical were enhanced by increasing *m*_*f*_ and introducing microchannels with 12.2% electrical efficiency.

Tyagi et al.^162^ experimentally examined a PVT module using a hybrid cooling approach involving MWCNT nanofluid for active cooling and a PCM for passive cooling. The impact of varying MWCNT concentrations (0.1–0.2%) and volumetric flow rates (0.5–1.5 L/min) of water and nanofluids as HT fluids is investigated. Results indicate that a *m*_*f*_ of 1.5 L/min yields optimal outcomes. The PV/T-PCM system with 0.2% MWCNT demonstrates the highest energetic and exergetic efficiencies, achieving 13.25%, 65%, 12.81%, and 1.66%, respectively.

Simón-Allué et al.^163^ experimentally explored the performance of various PVT panels and their energy efficiency after incorporating a layer of PCM within the panel. Different materials and designs were tested and compared with a traditional sheet-and-tube copper absorber to evaluate the energy performance of the heat absorber. Both glazed and unglazed configurations were examined for each model. The PVT panels were exposed to maximum solar irradiance and subjected to various operational conditions to assess their thermal performance thoroughly. Results indicated only minor variations in electrical output but a significant difference in thermal performance between the glazed and unglazed configurations. Among the heat absorbers, aluminum showed superior thermal performance compared to the polymeric option, although electrical output remained similar. Regarding the PCM, while there was no substantial improvement in thermal performance, it did lead to a more even distribution of heat, generating up to 30% of the maximum thermal power even after the removal of sun exposure. Although further research is required to understand the long-term effects of PCM on PV-thermal systems, this study offers valuable experimental data on the performance of PCM in conjunction with different heat absorber materials.

Jurčević et al.^164^ numerically investigated the robust performance of a new free-standing PVT collector equipped with integrated organic PCM under varying weather conditions. The research employed a novel approach integrating experimental data on an unconventional organic PCM, specifically pork fat, whose thermal properties (melting point, latent heat of melting) were determined using differential scanning calorimetry (DSC). The PCM’s melting process was observed to start at 8.3°C and conclude at 45.2°C, with a latent heat of 45.4 Jg^−1^.

Bhutto et al.^165^ experimentally studied lauric acid (organic PCMs) with Ag and Gr hybrid NPs used as additives. The results show that the LAG-3.5 nanocomposite, which consists of LA with 3.5% hybrid Ag and Gr NPs, achieved the highest reduction in transmissibility at 76%. This nanocomposite also improved the thermal conductivity of LA by 57% due to the high *k*_*p*_ of the hybrid NPs. Furthermore, the LAG-3.5 composite demonstrated an 8% increase in latent heat enthalpy. After 500 thermal cycles, LAG-3.5 maintained chemical stability and thermal durability. In terms of performance, the optimal LAG-3.5 composite exhibited improved electrical characteristics when used in PV and thermoelectric generator applications. This nano-enhanced composite shows significant potential for applications requiring a melting temperature range of 45°C–50°C.

Xu et al.^166^ experimentally studied the impact of hexadecanol-palmitic acid/expanded graphite (HA-PA/EG) eutectic composite PCM. When integrated into PV panels, HA-PA/EG effectively absorbs heat from solar radiation of 815.9 W/m^2^, reducing panel temperature by 5.1°C. These materials exhibit superior *kp*, leakage resistance, thermal stability, and cycling durability.

**Figure 21:**
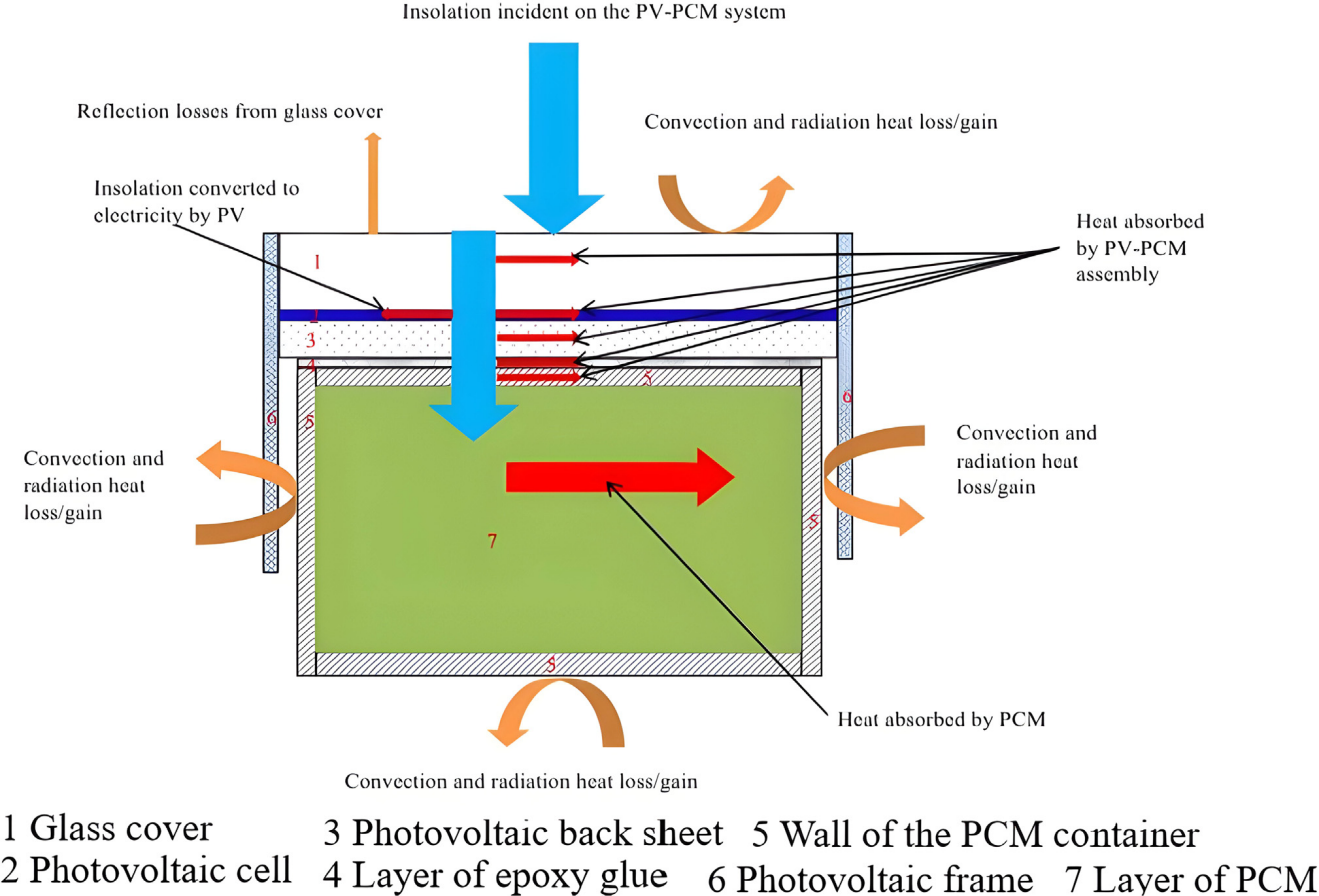
Schematic of PVT with PCM system Reproduced with permission^167^ Copyright 2015 Elsevier Ltd.

As depicted in Figure 21,^167^ Hasan et al.^167^ conducted an extensive investigation into PV-thermal systems coupled with PCM (PVT-PCM), deploying them in various geographical locations to evaluate their performance under diverse weather conditions. Their study proposed that PCM solidification would occur more rapidly in cooler regions like Ireland. In contrast, PCM would absorb larger heat loads in warmer climates like Pakistan but solidify slower. Both experimental and numerical outcomes show that calcium chloride hexahydrate outperformed capric-palmitic acid, achieving temperatures 3°C–4°C higher in Pakistan. Consequently, PVS deployed in warmer climates exhibited more significant power reserves owing to stable and higher radiation intensity.

### Nanofluids

**Figure 22:**
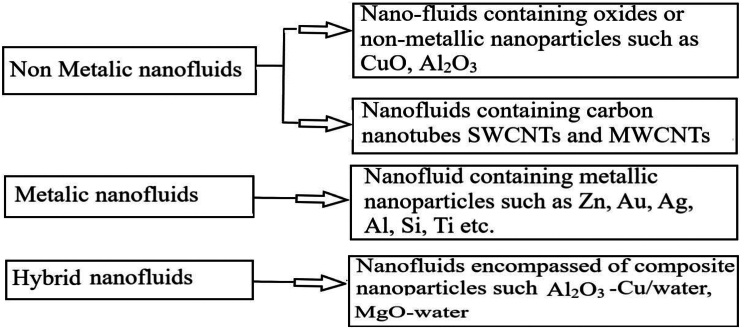
Various types of Nanofluid based on nanoparticle dispersion Reproduced with permission^171^ Copyright 2020 Elsevier Ltd.

The utilization of nanofluids in contemporary PVT setups has surged in popularity due to their advantageous attributes, such as heightened thermal conductivity and absorption of light.^168^ Studies have indicated that incorporating nanofluids as cooling agents in the thermal collectors of PVT setups ominously enhances efficiency by regulating the elevated temperatures of SC.^169^^,^^170^ The array of nanofluid variants is illustrated in Figure 22.^171^ A few of the nanofluid properties, along with the base fluid, are shown in Table 7.^148^

**Table 7:** Properties of Nanofluid with water as a base liquid at 298 K

Properties	Base fluid (H_2_O)	Al_2_O_3_ +H2O			CuO + H2O			TiO_2_ + water		
		0.4	0.8	1	0.4	0.8	1	0.4	0.8	1
ρ	998.2	1010	1021	1027	1020	1042	1053	1011	1024	1030
C_p_	4182	4128	4075	4050	4089	3999	3956	4123	4065	4037
*K*_*p*_	0.6	0.606	0.61	0.617	0.606	0.61	0.616	0.605	0.611	0.614
μ	0.001	0.00098	0.00021	0.001	0.0009	0.001	0.001	0.0002	0.0002	0.0002

NP is pivotal in HT effectively between the solid surface and fluid. Hence, choosing the nanofluid variant in a PVT setup is crucial to certify harmony and fulfill the anticipated enactment standards. Table 8 outlines research endeavors, including PVT setups with nanofluids as cooling agents, encompassing diverse types and arrangements.^70^^,^^172^^,^^173^^,^^174^^,^^175^^,^^176^^,^^177^^,^^178^^,^^179^^,^^180^^,^^181^^,^^182^

**Table 8:** Summary of research exploring nanofluid utilization in PVS

Authors	Types	Efficiencies (%)		Conclusions
		Electrical (%)	Thermal (%)	
Tian et al.^172^	MgO			Increased NP concentration improves output (exergy).
Menon et al.^173^ Experimental.	CuO	35.7	78.4	Nanofluid efficiently stores sensible heat during the PVT process as a cooling medium.
Bassam et al.^174^ Experimental.	SiC	10.8		SiC averts the development of microcracking but leads to a substantial decline in efficiency.
Ahmadinejad and Moosavi^70^	CuO + CNT	18.02	65.02	CNT exhibits superior results to CuO and distilled water under varying irradiation conditions.
Geovo et al.^175^ Theoretical.	MgO (1–1.5 vol. %)			Utilizing NP for cooling purposes has the potential to decrease the dimensions of STC
Wole et al.^176^ Experimental.	Al_2_O_3_-ZnO			Viscosity rises while specific heat declines upon increasing the vol. % of NP
Fayaz et al.^177^ Experimental.	MWCNT	12.6	81.2	Increasing *m*_*f*_ helps sustain temperature gradients, thereby improving *h*_*m*_.
Hooshmandzade et al.^178^ Experimental.	Hybrid (Al_2_O_3_ + SiO_2_) (0.1–0.5 %wt)		62.5	The implementation of nanofluids reduces temperature owing to their high *kp*, consequently boosting open-circuit voltage output.
Shen et al.^179^ Experimental.	ZnO (*m*_*f*_ = 0.008–0.012 kg/s)	9.08	33.9	Voltage creation exhibits a continuous increase over time, notwithstanding temperature fluctuations, which may initially rise before decreasing.
Murtadha^180^ Experimental.	Hybrid (Al_2_O_3_ + TiO_2_) ϕ = 2 wt %	17.6		The combination of Al_2_O_3_ with TiO_2_ surpasses standalone Al_2_O_3_ as an effective coolant.
Jasim et al.^181^ Numerical.	serpentine tube	31.1	5.4	Fe_3_O_4_-MWCNT with 1.5 vol. % produces maximum performance.
Miqdam et al.^182^	MWCNT	88.85	20	0.5 vol. % MWCNT

Tian et al.^172^ numerically studied the effect of the *m*_*f*_ of MgO/water in HT and the efficiency of 250 W PVT configuration. Results showed that a higher *m*_*f*_ of coolant leads to lower fluid temperature at the outlet, which increases exergy output. Exergy values are estimated based on the difference in outlet and ambient temperature. Maximum energy value is at a *m*_*f*_ of 0.5 L/min with an NP vol. % of 1.

Menon et al.^173^ emphasized the use of coolant in augmenting efficiencies (thermal and electrical) in PVS. Their outcomes demonstrated that utilizing nanofluid as a cooling agent increased electrical efficiency by 35.6% and 20.78% compared to PVT setups without cooling and those water-cooled. This enhancement is accredited to the superior *kp* of nanofluids over water, enabling more effective heat dissipation.

Bassam et al.^174^ devised PVT system using a combination of micro-fin tubes with twisted tape inserts with PCMs to augment thermal efficacy. Among different arrangements, the most efficient HT augmentation approach involved the utilization of micro-fin tubes with SiC nanofluids alongside nano PCMs. Under 800 W/m^2^ of *I*_*s*_, this setup achieved a thermal efficiency of 83.3% with an electrical efficiency of 10.8%.

Ahmadinejad and Moosavi^70^ examined the effectiveness of channeling systems with baffles in PVT setups. They observed that baffled channels facilitated a more efficient HT from SC to the coolant than the system without baffles. Outcomes showed that CNTs were superior in curbing the escalation of cell temperatures caused by the rise in solar irradiation compared to CuO or water. (Typically, heightened SC temperatures show a decline in the system’s electrical efficiency.).

Geovo et al.^175^ computational investigation on STC (flat-plate) utilizing MgO nanofluids, employing MATLAB, found that the highest thermal efficiency was recorded at 0.75vol. % of MgO NP, as higher concentrations diminishes efficiency.

Wole et al.^176^ studied the consequence of Al_2_O_3_-ZnO composite nanofluids ϕ on *C*_*p*_ and μ of water. Results indicate that μ rises as volume concentration increases while specific heat declines. Specifically, Al_2_O_3_-ZnO water composite nanofluids at a 2:1 blending ratio exhibit a maximum μ rise of 96.37%, a maximum *C*_*p*_ decline of 30.12% at 25°C, and a volume concentration of 1.67%.

Fayaz et al.^177^ delved into the influence of nanofluid’s varying *m*_*f*_ (30 L/h to 120 L/h) on PVT systems with constant solar radiation of 1000 W/m^2^. The result was obtained using experimental and numerical comparisons between water and MWCNT NP, which showed that the surface temperature of PVT changed with *m*_*f*_. Elevated nanofluid flow rates (at 60 L/h) showed a larger decline in temperatures at the outlet. The research highlighted the dominance of using MWCNT over water in several scenarios.

Hooshmandzade et al.^178^ implemented a PVS within and outside a greenhouse to check the impact of diverse environmental conditions on system performance. Three different types of coolant were used: pure water, Al_2_O_3_ nanofluid, SiO_2_, and a hybrid nanofluid (Al_2_O_3_ + SiO_2_). Results revealed that hybrid nanofluid improved system cooling performance, predominantly for indoor setups.

Shen et al.^179^ examined the efficacy of PVS utilized in hydrogen generation by electrolysis. Their research suggested that augmenting PVT efficiency with NP could counteract the reduction in hydrogen generation caused by decreased radiation, mainly when using larger coolant volumes. The best results were obtained by employing the highest volume of coolant fluid with 0.25 vol. % ZnO NP.

Murthada^180^ experimentally investigated the cooling of PV using NP to improve their efficiency, lifespan, and power output. A 2 wt % Al_2_O_3_/TiO_2_ hybrid NP was employed to assess its performance and compare it with previous studies that utilized Al_2_O_3_ and TiO_2_ nanofluids individually at the same concentrations.

The experimental setup consisted of three PV panels with identical specifications but different cooling methods in a single-pass flow configuration. Panel PV-1 was cooled with the 2 wt % Al_2_O_3_/TiO_2_ hybrid nanofluid, panel PV-2 used only water for cooling, and panel PV-3 operated without any cooling. The experiments were conducted at varying cooling fluid *m*_*f*_, ranging from 0.5 L/min to 3 L/min. The cooling performance (with NP) was compared against the reference panel PV-3, showing the maximum output powers of 46.6 W with hybrid nanofluid, 45.1 W for water cooling, and 41.9 W for the uncooled panel. This demonstrated an 11.2% increase in output power with the hybrid nanofluid compared to the uncooled panel. The temperature rise was 9.6°C with the hybrid nanofluid, compared to 8.4°C with water cooling, with electrical energy efficiency of 17.6%.

Jasim et al.^181^ numerically studied a novel geometry of serpentine tubes to enhance the performance of a PVT system. The investigation involves three hybrid nanofluids and two types of PCM tested within a serpentine tube of various cross-sectional (rectangular, circular, and triangular). The findings indicate that incorporating a PCM composite in rectangular cross-section serpentine tubes improved electrical and thermal efficiency by 5.4% and 31.1%, respectively, compared to configurations without PCM and with a circular cross-section. To further evaluate performance, the impact of three hybrid nanofluids at a 1.5% volume concentration was analyzed to determine which nanofluid enhances HT. Fe_3_O_4_-MWCNT was the most effective nanofluid for cooling the SC temperature. When Fe_3_O_4_-MWCNT was used at a volume concentration of up to 4.5%, it significantly affected vital parameters such as cell temperature, fluid outlet temperature, and electrical and thermal efficiency. Additionally, combining Fe_3_O_4_-MWCNT (4.5%) with PCM and PCM composite increased exergy efficiency by 13.3% and 16.2%, respectively.

Miqdam et al.^173^ used MWCNT as NP in a PVT system. The effect of constraints such as the base fluid (ethylene glycol, propylene glycol, and HT oil), surfactant, and sonication duration used during mixing was investigated. The result indicates that 0.5 vol. % MWCNT with water as the base fluid enhanced *kp* by 119.5%, 308%, and 210% for ethylene glycol, propylene glycol, and HT oil. Moreover, the PVT system cooled by this nanofluid demonstrated an electrical efficiency 88.85% higher than PVT and 44% higher than water-cooled PVT systems. Furthermore, its thermal efficiency was 20% greater than water-cooled PVT systems.

Nonetheless, despite the potential for enhancing heat transfer, the utilization of nanofluids poses several hurdles, including diminished *Cp*, stability challenges, heightened *Δp*, foam generation, augmented μ, and escalated expenses.^183^ Adequate preparation is indispensable in tackling these obstacles. Surfactants are commonly employed in the preparation process to stabilize nanofluids, thereby mitigating or enhancing stability and diminishing surface tension. Nevertheless, the use of surfactants may also detrimentally impact the *k*_*p*_ of the nanofluid, resulting in diminished efficacy.^184^ Pressure drop concerns in nanofluids frequently stem from amplified concentration (vol. %), which is associated with μ.^185^ Moreover, larger *f* can lead to heightened Δ*p*,^186^ necessitating greater pumping power to facilitate fluid circulation during operation.^187^ Consequently, maintaining consistent pumping power becomes imperative to sustain ideal performance.

## Comparison among cooling methods

Previously stated in this document are five distinct approaches to address the issue of overheating PV systems while simultaneously enhancing their performance. Despite sharing common development objectives, each method possesses unique perspectives and hurdles. Table 9 delineates the merits and demerits of these methodologies, facilitating a comparative analysis for improving PV efficiency. Such insights can heighten the awareness of auspicious avenues and provide a foundation for future advancements.

**Table 9:** Merits/demerits of diverse PVT systems

PVT System	Advantages	Disadvantages
Absorber design	• Easily installed within conventional PV • Higher efficiency • All absorbed heat can be salvaged • Ease of modification	• High installation charges • Requires external power for circulating cooling liquid • Non-uniform cooling generates a hotspot • larger installation space required
Mini/microchannels	• Uniform distribution of temperature • Compact in design • Efficiency	• Requires higher pumping power • Mostly applicable for concentrated PV • Higher installation/maintenance cost
Polymers	• Low initial cost • Ease in fabrication • Corrosion resistance • generally Lightweight	• Low HT augmentation • Productive upon combining with other techniques • Absorbed heat cant be reused
PCM	• Fluid circulation is not needed. • Easily attach • Low manufacturing cost • absorbs extra heat	• Direct physical contact with PV material can lead to damage. • leakage risk is higher • Degradation upon prolonged exposure
Nanofluids	• Significant efficiency enhancement • Low manufacturing and maintenance cost • Ease of integration with other cooling methods	• Additional pumping power required • Additional HX required • Nanofluid accumulation and its stability are primary concerns

Redesigning the absorber component to optimize the system offers notable advantages in manufacturing flexibility, as collector designs can be tailored in various ways. However, the efficacy of this system could be further improved pending resolution of the high fabrication costs. Challenges pertaining to absorber design, particularly regarding space requirements, may be resolved through mini-microchannel technology, which augments heat transfer area without increasing spatial demands. Nonetheless, using small-diameter hydraulic pipes entails increased pumping power, necessitating careful diameter adjustments to achieve maximal performance while minimizing power consumption.

Additionally, employing polymer materials as collector components presents a cost-effective alternative to metal counterparts. Despite the comparatively modest performance levels achieved, integrating polymer materials with other methodologies remains a promising avenue for advancement. Another viable option involves leveraging PCMs, which exhibit superior heat absorption properties. However, the efficacy of PCM-based solutions is contingent upon the melting point, with performance dilapidation over time attributed to exposure.

Among the five approaches, the nanofluid method stands out for its ability to attain the highest electrical efficiency. Furthermore, its simplicity of application and cost-effectiveness underscore its significant potential for enhancement. The primary challenge associated with this method lies in mitigating the escalating pumping power that accompanies higher concentrations of nanofluids.

## Conclusions

To combat overheating in PV systems, the evolution of PVT systems has spanned decades, incorporating various technologies to align with market needs. This article delves into five primary strategies: polymer materials, redesigned absorbers, nanofluids mini/microchannels, and PCMs. These techniques strive to reduce solar cell temperatures by optimizing heat absorption within the absorber using different approaches. Their effects on thermal and electrical performance are assessed, yielding several noteworthy conclusions.

• Absorber design: optimal PVT performance hinges on expanding heat transfer contact areas, achievable through elongated collectors, strategic placement of riser tubes, fluid flow homogenization using fins, and optimizing pipe arrangements to minimize flow distance.
• Mini/microchannels: despite enhancing heat transfer due to their diminutive size, mini/microchannels pose a risk of increased power loss. Modifications primarily focus on grooved external channels or channel pipe arrays, utilizing plain and adjusting diameters.
• Polymer materials: as an alternative to costly metal-based PVT systems, polymers offer design flexibility to maximize heat transfer via increased contact areas. They can replace glass covers as well. Although they have yet to surpass metal-based systems in cost-effectiveness, performance and adaptability, they promise future advancement.
• PCMs: with their latent heat storage capability, PCMs efficiently absorb heat. Their integration efficiency depends on factors like thickness, type, and placement. PCM integration can offer more effective heat absorption than altering absorber materials.
• Nanofluids: nanofluids, with superior thermal conductivity compared to water, show promise as coolants. Higher vol. % concentrations generally result in better heat dissipation and heightened electrical efficiency. Nonetheless, their preparation methods influence nanofluids’ stability and heat absorption.

These findings offer valuable visions into the ongoing development of PVT systems and underscore possible avenues for future exploration and innovation. The five cooling systems studied, absorber design, mini/microchannels, and polymer materials, demonstrate notable performance optimization by augmenting HT areas or manipulating HT processes between the coolant medium and collector. However, a trade-off exists between fabrication costs and the level of improvement.

### Limitations of the study

PCMs and nanofluids present significant and cost-effective enhancements, but their limitations must be addressed. Moreover, conducting life cycle assessment analyses is imperative for comprehending the sustainability of these techniques, particularly concerning economic, manufacturing, and environmental aspects such as energy payback time, solid waste generation, and carbon footprint reduction.

## Resources availability

### Lead contact

Further information and requests for resources and reagents should be directed to and will be fulfilled by the Lead Contact, S M Mozammil Hasnain (E-mail: smmh.429@gmail.com).

### Materials availability

This study did not generate new unique reagents. Requests for resources and reagents should be directed to and will be fulfilled by the lead contact, S M Mozammil Hasnain (E-mail: smmh.429@gmail.com).

### Data and code availability

• Any additional information required to reanalyze the data reported in this paper is available from the lead contact upon request.

## Acknowledgments

This work was supported by the Kazan Federal University Strategic Academic Leadership Program (PRIORITY-2030).

## Author contributions

Md.A.R.: conceptualization, methodology, software, formal analysis, resources, writing—original draft preparation, writing—review and editing. S.K.G.: data collection and writing—review and editing. N.A.: methodology, writing—review and editing. R.Zhapparbergenov: conceptualization, software, supervision, writing—review and editing. S.M.M.H.: conceptualization, writing—review and editing, writing—original draft preparation. R.Zairov: supervising, writing—review and editing.

## Declaration of interests

The authors declare no competing interests.
